# Supplementary material for: The transmission of pottery technology among prehistoric European hunter-gatherers
Source: Nat Hum Behav. 2022 Dec 22;7(2):171–83. doi: 10.1038/s41562-022-01491-8 (PMC9957732; doi:10.1038/s41562-022-01491-8)
Supplement: Supplementary file 1 — Supplementary Methods, Results, Figs. 1–12, Tables 1–12 and References. [file 41562_2022_1491_MOESM1_ESM.pdf]

nature human behaviour

Article

https://doi.org/10.1038/s41562-022-01491-8

The transmission of pottery technology among prehistoric European hunter-gatherers

In the format provided by the authors and unedited

Contents

Supplementary Methods **3**

Background to dating and spatiotemporal regression models 3

Site chronologies: methods 3

Estimating diffusion rate 4

Predicting arrival time 4

Phylogenetic analyses 5

Spatial modelling of the Δ13C values 5

Supplementary Results **6**

Site chronology for Kairshak III 6

Site chronology for Baibek 6

Site chronology for Algay 6

Site chronology for Cherkasskaya 5 7

Site chronology for Rakushechny Yar 7

Site chronology for Zamostje 2 8

Site chronology for Zvidze 8

Spatial temporal regression results 9

Mantel test results on regional subsets of the data 9

Mantel test results for strictly 'Early Neolithic' vessels 10

Phylogenetic analyses 10

Supplementary Figures **11**

Supplementary Tables **23**

Supplementary Table 1: Sites in the study 23

Supplementary Table 2: New and/or previously unpublished 14C results 29

Supplementary Table 3: previously-published 14C ages used in chronological models 36

Supplementary Table 4: Estimated average rates of transmission 39

Supplementary Table 5: Modelled front speeds in archaeological case studies 40

Supplementary Table 6: Pottery traits 40

Supplementary Table 7: 95% confidence interval for Mantel correlation coefficients and associated p-values 47

Supplementary Table 8: Distribution of Jaccard distances (interquartile range) 48

Supplementary Table 8: Mantel test coefficients with vessels as the unit of analysis 48

Supplementary Table 9: Mantel test coefficients limited to major regions 49

Supplementary Table 10 : Mantel test coefficients stratified by vegetation zone 49

Supplementary Table 11 : Mantel test coefficients for earliest forms only 50

Supplementary References **51**

Supplementary Methods

Background to dating and spatiotemporal regression models

The methods we use to measure diffusion rate and make predictions for the arrival time of pottery follow the results of Silva and Steele [1,2], who demonstrated that distance measurements along least cost paths produce a spatio-temporal regression model that fits the archaeological data very in the case of the spread of the Neolithic across Western Europe. Jordan et al [3] used similar techniques to examine early pottery use across the entire Afro-Eurasian landmass, where dated occurrences of pottery from across the landmass were used to predict an 'arrival time' for any point in the landscape. Our study differs from previous work because we combine prior information and radiocarbon dates in Bayesian models of the 'start' date for the early phase of pottery use at a small number sites where radiocarbon measurements from samples of atmospheric carbon (i.e. without the complicating effects of freshwater carbon reservoirs) can be stratigraphically associated with early pottery. The posterior probability distribution of the 'start' of pottery at each site is then used to parameterize a regression between distance and time. The distance between each site and a putative origin is determined using a 'least cost path' (LCP) algorithm (details of the script used are given digitally in the electronic repository of code and data; see doi.org/10.5281/zenodo.6619101). The utility of this approach depends on how the modelling process plays out. If the model and data fit, we should be able to see a straight-line regression between time and distance, implying that there is a wave front moving across the landscape that carries with it the knowledge of pottery manufacture. We can gain multiple perspectives on the wavefront by considering each site as an origin point where earlier sites have 'negative' distance, and later sites a 'positive' distance. This removes any dependence on an absolute point of origin for the study region, which in any case is rather moot as the traditions can be traced back to the Far East.

Site chronologies: methods

To anchor the distance-time regression, we estimate the date of the first pottery at seven sites between the Caspian and the Baltic, using simple Bayesian chronological models implemented in OxCal 4.4 [4]. These models (Supplementary Figures 1–7) use only calibrated ^{14}C dates from fully terrestrial species (herbivore bones, and occasionally wood charcoal or plant macrofossils). The main interpretative challenge, when relying on dates of bones and plant remains, is assessing whether pottery was exactly contemporaneous with these materials, particularly when the dates of terrestrial samples span several centuries. Each site chronology depends on at least 9 calibrated dates, which is enough to allow detection of outliers if the occupation was short-lived. In one case, Cherkasskaya 5, our model suggests that an equid tooth, precisely dated by two laboratories using accelerator mass spectrometry (AMS), is 100–200 years older than the other 8 samples, and can be omitted from the phase of occupation in which pottery was deposited. Elsewhere, the relevance of less precise early radiometric dates on terrestrial material is more ambiguous (see Kairshak III, Algay). Nevertheless, the seven sites discussed here are those with the most robust ^{14}C chronologies, if not necessarily the oldest sites with pottery in each region. Each model's start Boundary provides a probability distribution for the date at which pottery first appeared at that site.

Estimating diffusion rate

We undertook linear regression between distance and time using several methods to obtain a range of plausible values for the rate of diffusion of pottery. Distance measurements are the length of the least-cost path between these locations, derived from the analysis of a 100m digital elevation model of Eurasia, the ASTER Global DEM v3 [23], using the *r.cost* and *r.drain* algorithms in GRASS GIS [24]. Using the scripts presented elsewhere in the supplementary information we use both reduced major axis regression (RMA) and ordinary least-squares (OLS) regression to undertake the analysis, employing the R *lmodel2* package for RMA [25].

Predicting arrival time

To use this modelling approach to predict arrival time, we then set up a grid over the landscape and run the least cost path analysis again from each point in that grid, and extrapolate through these results using a thin plate spline regression in the GIS. This produces a raster containing an

estimate of the cost distance for each point in the landscape. Next using straightforward raster algebra, can be parameterized with the results of the space time regression:

$$T = T_0 + D/r$$

Where T is the raster of predicted arrival dates, T_0 is the date at the point of origin, r is the gradient of the diffusion rate regression in km yr^{-1}, D is the cost-distance raster generated by the thin plate spline regression, equal to zero at the point of origin. The resulting surface T contains an array of pixels, each one predicting the year we could expect pottery to first occur. The terms T_0 and r have associated uncertainty that can be used to generate an error map using the same process.

Phylogenetic analyses

To investigate what kind of historic signals might be embedded in the pottery trait data, we applied phylogenetic network analysis [33]. Our goal here was to determine whether the data represent evolutionary patterns of branching, where cultural traits gradually differentiate, or blending, where different traits merge and recombine to form culturally recognisable entities. We were also interested in whether there was any empirical basis for inferring cladal relationships between site assemblages that have been well dated. Whilst the full dataset is too large to analyse in this manner, we employed from the eight sites where the pottery arrival times were more robustly established.

Spatial modelling of the $\Delta^{13}C$ values

A local average model (AverageR) available in *IsoMemo 1.9.13* (available from https://www.isomemoapp.com/), developed within the IsoMemo and Pandora initiative, was used to generate a spatial modelling of the $\Delta^{13}C$ values. AverageR is a generalised additive mixed model that uses a thin plate regression spline [34]. The model used for each sample the $\Delta^{13}C$ ($C_{18:0}$-$C_{16:0}$) values, the combined uncertainties of the isotopic measurement and the site attribution used as a random intercept to capture dependencies within one site.

Supplementary Results

Site chronology for Kairshak III

Kairshak III is one of several sites in the North Caspian plain, close to the mouth of the River Volga, where early Neolithic 'Kairshakskaya'-type pottery has been found. Seven herbivore bones (mainly kulan, *Equus hemionus kulan*), were newly dated (Supplementary Table 2) to complement the 4 legacy bone dates (Supplementary Table 3). All 8 AMS ^{14}C dates on animal bone, and 2 of the 3 radiometric bone dates, are compatible with a brief occupation episode in the early 6th millennium cal BC (Supplementary Figure 1). One radiometric bone date is a statistical outlier and has been excluded from the chronological model, which suggests that pottery only appeared here shortly after 5900 cal BC, notwithstanding a number of ^{14}C ages on the total organic carbon content (TOCC) of pottery, which when calibrated correspond to dates in the mid-7th millennium (Meadows, Dolbunova et al. in prep.).

Site chronology for Baibek

Recent excavations at Baibek, another site with Kairshakskaya pottery, revealed several structures attributed to a single phase of occupation [5]. All 32 of the newly obtained AMS ^{14}C dates on herbivore bones (Supplementary Table 2) are compatible with a brief occupation at around 5950 or 5900 cal BC (Supplementary Figure 2). Nine of the ten legacy radiometric dates on bones are consistent with this chronology, and one appears to be marginally later. Bulk charcoal radiometric dates also fit the short chronology. Food crust and TOCC ^{14}C ages corresponding to calibrated dates in the late 7th millennium are therefore assumed to be misleading (Meadows, Dolbunova et al. in prep.).

Site chronology for Algay

Five new AMS dates on herbivore bones (Supplementary Table 2) and 12 legacy dates on bones and charcoals (Supplementary Table 3), including a stratified sequence of 9 samples and an AMS

date on a horse bone (Supplementary Figure 3), span several centuries in the mid-late 6th millennium. Three radiometric dates on unidentified bones appear to fall in the later 7th and/or earlier 6th millennia. A similar situation, in which one or more radiometric dates on terrestrial material appear to be centuries earlier than a much larger number of AMS dates, also occurs at Varfolomeevka and Oroshemoe, the other two sites with early Neolithic Orlovskaya-type pottery and significant numbers of ^{14}C dates (Meadows, Dolbunova et al. in prep.). Our Algay model (Supplementary Supplementary Figure 3) disregards these early dates, which are not corroborated by stratigraphic sequences [6] and which are identified as outliers using OxCal's Outlier_Model functions [7], presumably either because the dates are inaccurate, or because the bones are not associated with the main period of occupation. Pottery appeared at Algay at a depth of 147–130cm, at the base of a stratigraphic sequence also dated by SPb-1510 [8].

Site chronology for Cherkasskaya 5

The chronological model for the brief early Neolithic occupation at Cherkasskaya 5, on the Middle Don, is based on AMS dates for 9 herbivore bones or teeth (Supplementary Table 3). One of these is a statistical outlier and is regarded as residual, leading to an estimated date for the appearance of pottery in the 59th century cal BC [10] (Supplementary Figure 4).

Site chronology for Rakushechny Yar

Rakushechny Yar, on the Lower Don, was long regarded as an importantly early site for Neolithic pottery in Eastern Europe, mainly on the basis of food-crust dates that are almost certainly misleadingly early, due to the persistent use of pottery to process fish and other aquatic species [11]. Out of the 50 bones or teeth of terrestrial herbivores that have now been dated from layers with the earliest pottery (Supplementary Tables 2 and 3), only one (SPb-729, 7970±110 BP, 7190–6590 cal BC) is necessarily older than c.5600 cal BC, and it is so much older that we do not regard it as relevant to the chronology of early pottery (Supplementary Figure 5). A more detailed chronological model, which also includes legacy ^{14}C data and results from new samples with ^{14}C reservoir effects, is in preparation.

Site chronology for Zamostje 2

Well over 100 ^{14}C ages are available from the waterlogged site, Zamostje 2, spanning three late-final aceramic Mesolithic layers and Early (Upper Volga pottery) and Middle (Lyalovskaya pottery) Neolithic layers. A large proportion of these are radiometric dates for vertical timbers (which are not associated stratigraphically with a specific layer) or bulk organic sediment (which may have contained redeposited, intrusive or aquatic plants). Many of the AMS dates are on pottery food-crusts, some of which have been shown to contain aquatic ingredients [12] and thus to be misleadingly old [13]. Several horizontally bedded fish traps and wood fragments, and charred plant macrofossils attached to 3 undecorated sherds of Upper Volga pottery (Supplementary Tables 2 and 3) provide enough reliable dates to date the start of the (surviving) Early Neolithic layer to the 56th century cal BC (Supplementary Figure 6). A more detailed chronological model, incorporating all known ^{14}C ages, is in preparation.

Site chronology for Zvidze

At Zvidze, the AMS date of an alder (*Alnus* sp.) charcoal fragment embedded in a potsherd from the earliest Neolithic layer provides a terminus post quem for that sherd, and implicitly for the start of pottery production locally [14] (Supplementary Table 3). Although animal bones from the 1980s excavations are preserved, it was not possible to assign them unambiguously to Mesolithic or Neolithic contexts. However, the legacy radiometric dates on wood and peat [15,16] (Supplementary Table 3) are compatible with the stratigraphic sequence [17] and with the AMS date for the potsherd. The resulting chronological model (Supplementary Figure 7) supports the view that pottery first appeared in eastern Latvia by c.5400 cal BC.

Spatial temporal regression results

Regressions were run multiple times using different samples of the posterior probability distributions of the chronological model of the start date at each of the sites in order to fairly incorporate chronological uncertainty (Supplementary Figure 8). RMA models produced slightly

faster diffusion rates than OLS models (Supplementary Figure 9). The modelled diffusion rates from both methods are given in Supplementary Table 4.

An obvious limitation of this analysis is that when sites are excavated, we cannot necessarily expect to find in every case the earliest, or even a relatively early, occurrence of pottery for each locality. This is especially true if pottery forms are used for a long time in each locale. We have at least one site – Rakushechny Yar – which does not really fit a diffusion model of the spread of pottery into the region. This is to be expected for some sites and this gives us new insight into the site itself and a new way to interpret its chronology.

Compared to other modelled or measured cases of archaeological diffusion, we find that the spread of early hunter-gatherer pottery in Europe was a rapid movement, up to 10km per year. This is much faster, for example, than the spread of pottery in Central-Western Europe, as various different studies using a selection of methodologies have shown (Supplementary Table 5).

Mantel test results on regional subsets of the data

In the southwest of our study area, around the northern shores of the Caspian Sea and in the catchments of the Don and Volga Rivers, the organic residues are indeed strongly correlated with technology, morphology and decoration. Moving westwards, pottery finds from the Upper Dnieper and Bug-Dniester plains (modern-day Ukraine and southern Belarus) have weaker correlations but these are still significant, aside from the absence of any significant relationship between use and decoration (Supplementary Table 9). In the northern regions, strong correlations are observed across the datasets, albeit weakly in the case of residues and decoration in the north-eastern part of our study area. Sites in the Baltic and across the western half of the study area show correlations between geographic distance and technology, which is absent in the east half, perhaps reflecting a more homologous pattern of manufacturing traditions such as the characteristic use of shell temper for Narva pottery and organic tempers for Neman pottery for example. Correlations between technology, morphology, decoration and use also present themselves when the sample is stratified by vegetation zone; in Taiga the smaller number of sites

and pottery finds reduces the statistical power of the sample but a significant correlation is nonetheless observed between morphology and organic residues (Supplementary Table 10). The small number of sites in desert biomes precludes undertaking this analysis separately for them.

Mantel test results for strictly 'Early Neolithic' vessels

We also performed the analysis on a subset of the data involving pots (917 vessels) that are identifiably 'Early Neolithic', i.e. excluding later/developed forms and also the various cultures of the western Baltic. The results are similar to the overall dataset (Supplementary Table 11). Using a partial Mantel test, a combination of all pottery traits against residues, holding distance constant, produces a Mantel r of 0.29 (95% C.I. 0.25 to 0.32, p ~ 0.001).

Supplementary Figures

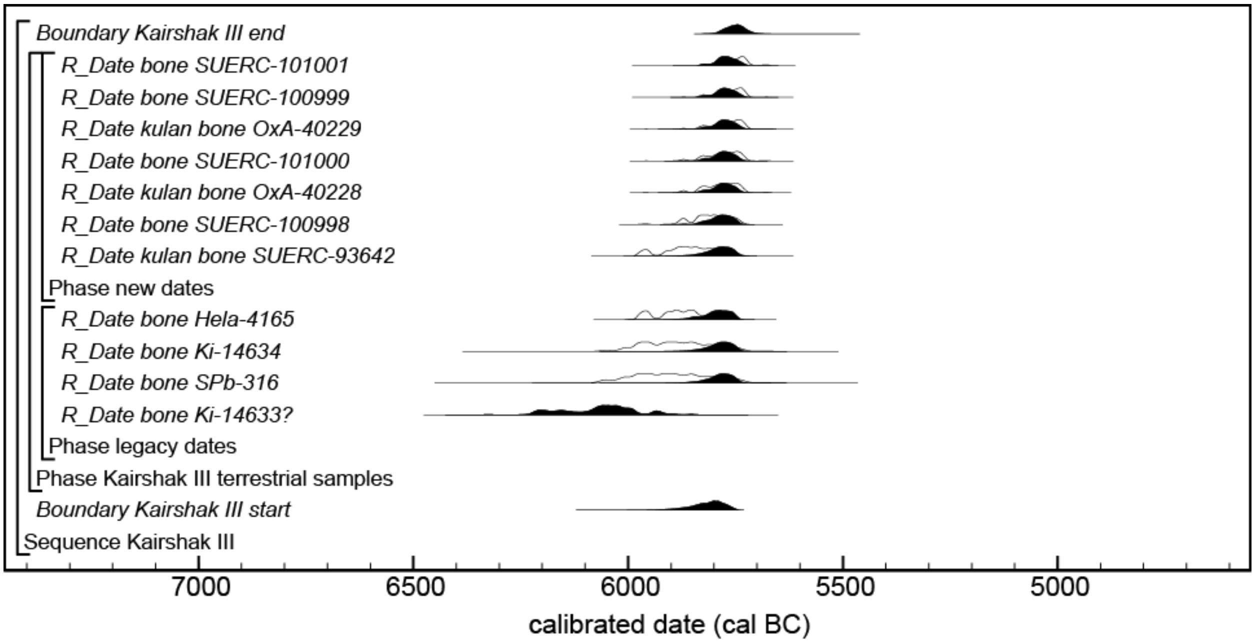

Supplementary Figure 1: Chronological model of the early Neolithic occupation of Kairshak III, showing two probability distributions for the calendar date of each sample: in outline, that obtained by

simple calibration of the ^{14}C age, using the IntCal20 calibration curve (Reimer et al. 2020); in black, the model's posterior density estimate of the sample date. Ki-14633 is omitted from the model (as indicated by '?'), and only the simple calibration (black distribution) of this result is shown. The model structure assumes that the dated samples represent a single, uniform phase of deposition with start and end boundaries, whose dates are calculated based on the scatter of calibrated dates (Bronk Ramsey 2009a). The boundary *Kairshak III start* is regarded as the date of the first pottery at this site.

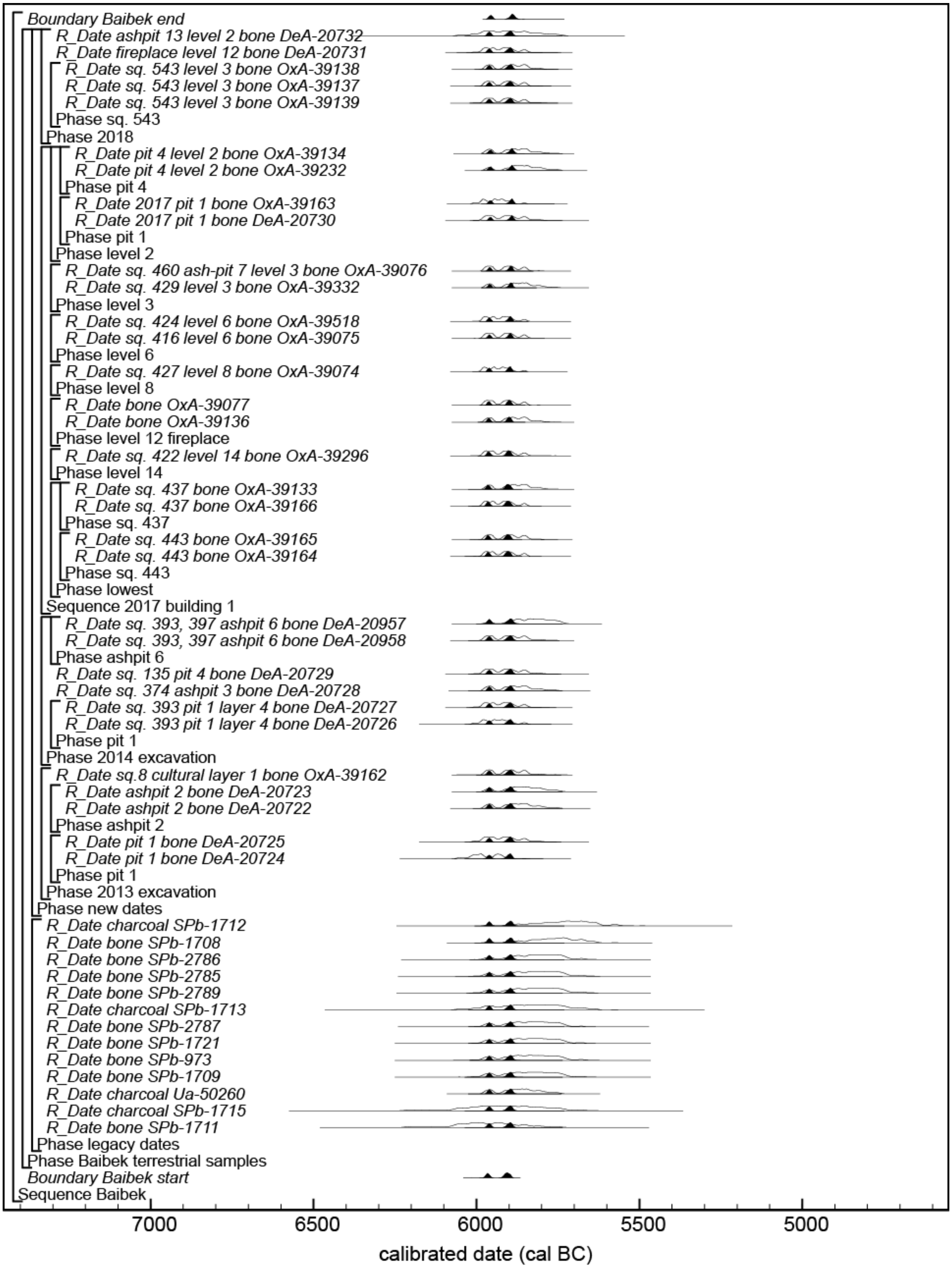
Boundary Baibek end
R_Date ashpit 13 level 2 bone DeA-20732
R_Date fireplace level 12 bone DeA-20731
R_Date sq. 543 level 3 bone OxA-39138
R_Date sq. 543 level 3 bone OxA-39137
R_Date sq. 543 level 3 bone OxA-39139
Phase sq. 543
Phase 2018
R_Date pit 4 level 2 bone OxA-39134
R_Date pit 4 level 2 bone OxA-39232
Phase pit 4
R_Date 2017 pit 1 bone OxA-39163
R_Date 2017 pit 1 bone DeA-20730
Phase pit 1
Phase level 2
R_Date sq. 460 ash-pit 7 level 3 bone OxA-39076
R_Date sq. 429 level 3 bone OxA-39332
Phase level 3
R_Date sq. 424 level 6 bone OxA-39518
R_Date sq. 416 level 6 bone OxA-39075
Phase level 6
R_Date sq. 427 level 8 bone OxA-39074
Phase level 8
R_Date bone OxA-39077
R_Date bone OxA-39136
Phase level 12 fireplace
R_Date sq. 422 level 14 bone OxA-39296
Phase level 14
R_Date sq. 437 bone OxA-39133
R_Date sq. 437 bone OxA-39166
Phase sq. 437
R_Date sq. 443 bone OxA-39165
R_Date sq. 443 bone OxA-39164
Phase sq. 443
Phase lowest
Sequence 2017 building 1
R_Date sq. 393, 397 ashpit 6 bone DeA-20957
R_Date sq. 393, 397 ashpit 6 bone DeA-20958
Phase ashpit 6
R_Date sq. 135 pit 4 bone DeA-20729
R_Date sq. 374 ashpit 3 bone DeA-20728
R_Date sq. 393 pit 1 layer 4 bone DeA-20727
R_Date sq. 393 pit 1 layer 4 bone DeA-20726
Phase pit 1
Phase 2014 excavation
R_Date sq.8 cultural layer 1 bone OxA-39162
R_Date ashpit 2 bone DeA-20723
R_Date ashpit 2 bone DeA-20722
Phase ashpit 2
R_Date pit 1 bone DeA-20725
R_Date pit 1 bone DeA-20724
Phase pit 1
Phase 2013 excavation
Phase new dates
R_Date charcoal SPb-1712
R_Date bone SPb-1708
R_Date bone SPb-2786
R_Date bone SPb-2785
R_Date bone SPb-2789
R_Date charcoal SPb-1713
R_Date bone SPb-2787
R_Date bone SPb-1721
R_Date bone SPb-973
R_Date bone SPb-1709
R_Date charcoal Ua-50260
R_Date charcoal SPb-1715
R_Date bone SPb-1711
Phase legacy dates
Phase Baibek terrestrial samples
Boundary Baibek start
Sequence Baibek
7000
6500
6000
5500
5000
calibrated date (cal BC)

(previous page) **Supplementary Figure 2**: Chronological model of the early Neolithic occupation of Baibek, with the same format as Supplementary Figure 1. SPb-1708 and SPb-1712 are omitted from the model, as they are appreciably later than the rest of the results. The stratigraphic sequence in building 1 of the 2017 excavation [5] is used to order the dates of samples concerned. Duplicate 14C measurements on the same skeletal element have been combined before calibration.

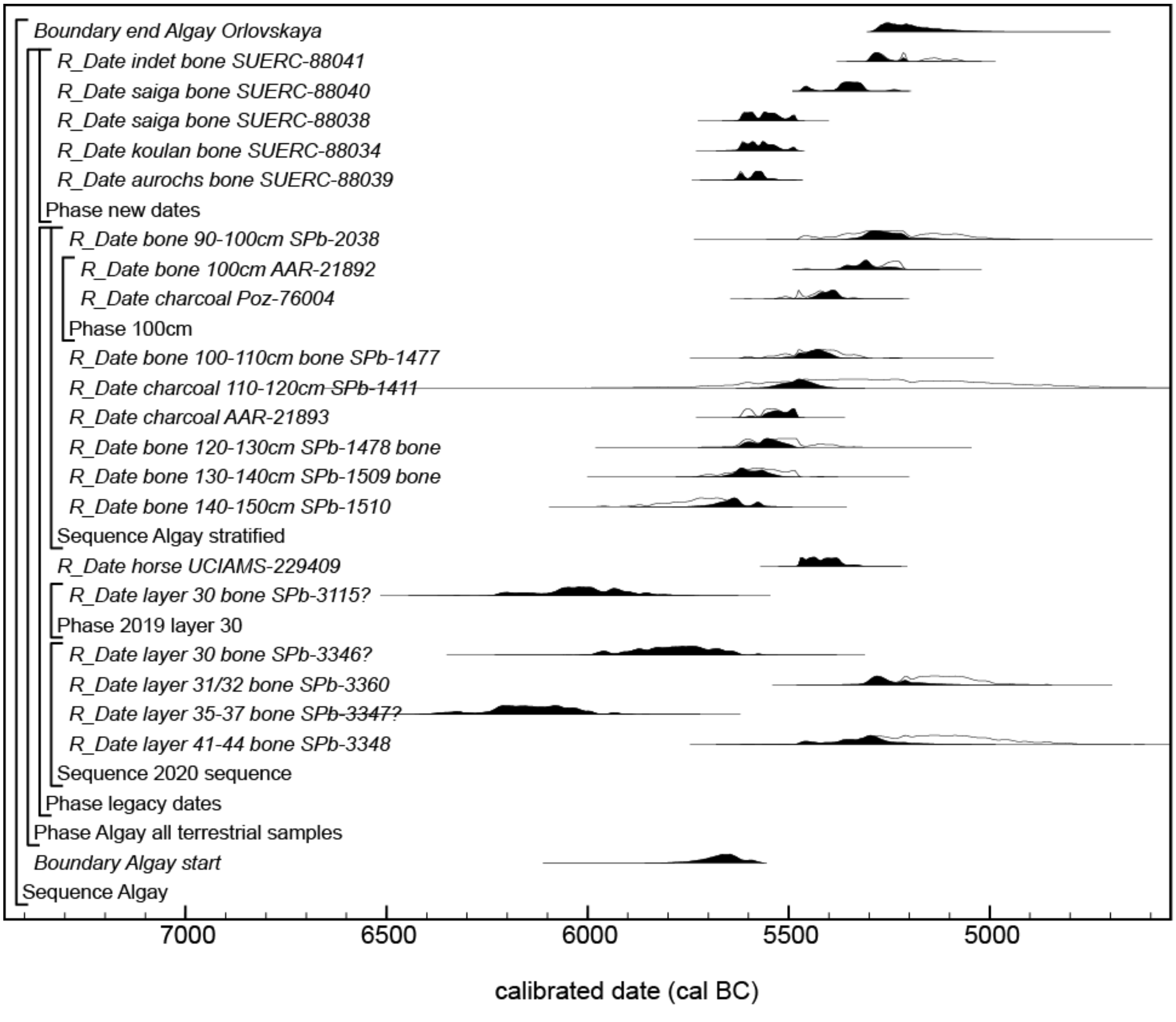

Supplementary Figure 3: Chronological model of the early Neolithic occupation of Algay, with the same format as Supplementary Figure 1. SPb-3115, SPb-3346 and SPb-3347 are omitted from the model, as they appear to be significantly earlier than other results, with unsatisfactory individual indices of agreement (Bronk Ramsey 2009a). The stratigraphic sequence is used to order the dates of samples concerned [9].

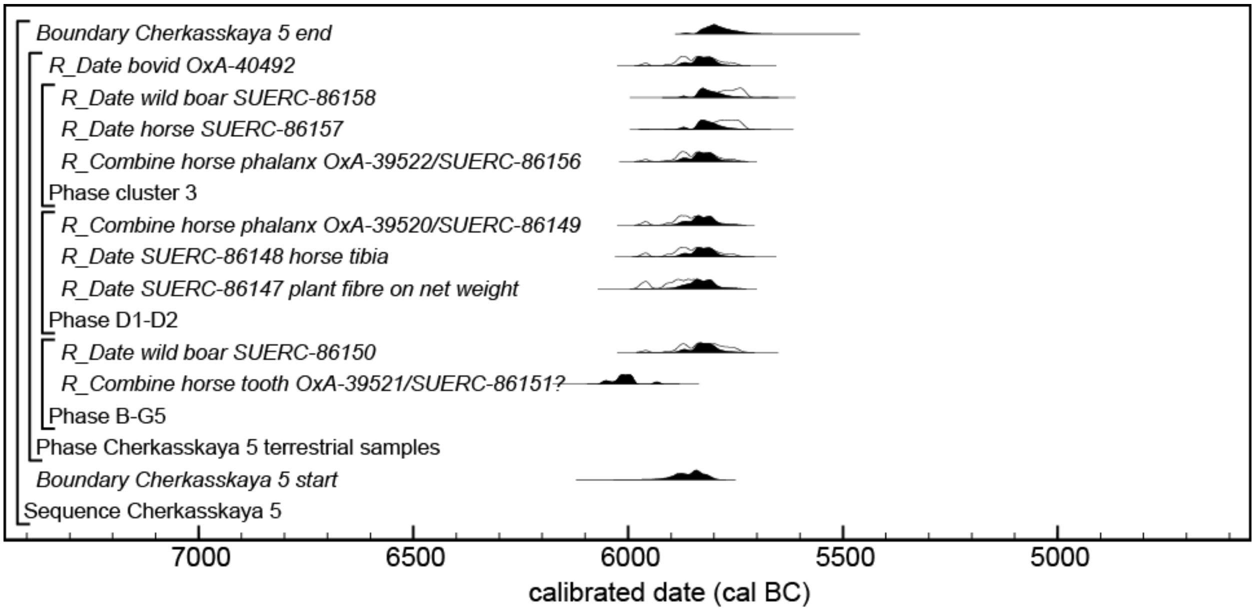

Supplementary Figure 4: Chronological model of the early Neolithic occupation of Cherkasskaya 5, with the same format as Supplementary Figure 1. Duplicate 14C measurements on the same skeletal element have been combined before calibration.

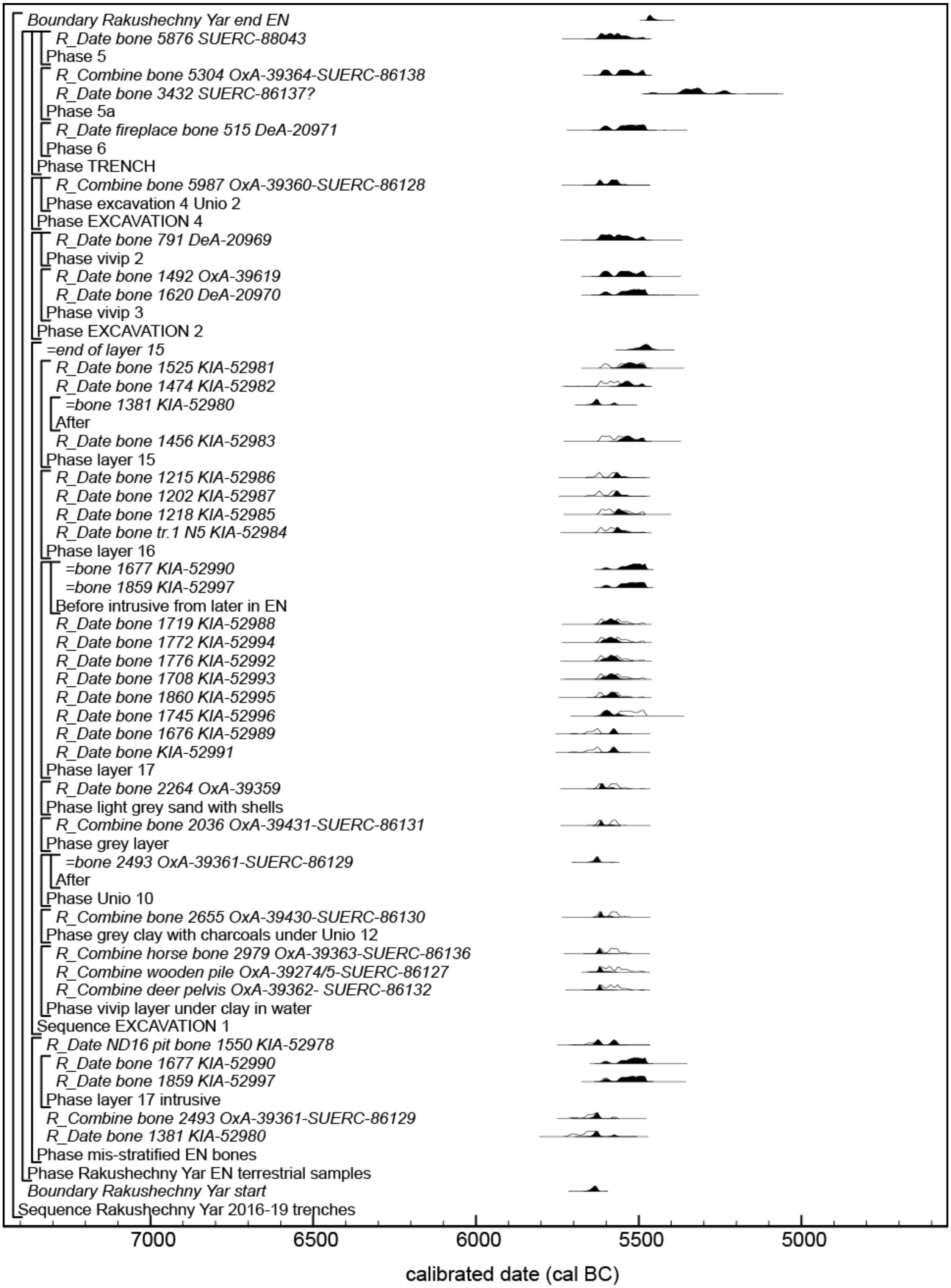
Boundary Rakushechny Yar end EN
R_Date bone 5876 SUERC-88043
Phase 5
R_Combine bone 5304 OxA-39364-SUERC-86138
R_Date bone 3432 SUERC-86137?
Phase 5a
R_Date fireplace bone 515 DeA-20971
Phase 6
Phase TRENCH
R_Combine bone 5987 OxA-39360-SUERC-86128
Phase excavation 4 Unio 2
Phase EXCAVATION 4
R_Date bone 791 DeA-20969
Phase vivip 2
R_Date bone 1492 OxA-39619
R_Date bone 1620 DeA-20970
Phase vivip 3
Phase EXCAVATION 2
=end of layer 15
R_Date bone 1525 KIA-52981
R_Date bone 1474 KIA-52982
=bone 1381 KIA-52980
After
R_Date bone 1456 KIA-52983
Phase layer 15
R_Date bone 1215 KIA-52986
R_Date bone 1202 KIA-52987
R_Date bone 1218 KIA-52985
R_Date bone tr.1 N5 KIA-52984
Phase layer 16
=bone 1677 KIA-52990
=bone 1859 KIA-52997
Before intrusive from later in EN
R_Date bone 1719 KIA-52988
R_Date bone 1772 KIA-52994
R_Date bone 1776 KIA-52992
R_Date bone 1708 KIA-52993
R_Date bone 1860 KIA-52995
R_Date bone 1745 KIA-52996
R_Date bone 1676 KIA-52989
R_Date bone KIA-52991
Phase layer 17
R_Date bone 2264 OxA-39359
Phase light grey sand with shells
R_Combine bone 2036 OxA-39431-SUERC-86131
Phase grey layer
=bone 2493 OxA-39361-SUERC-86129
After
Phase Unio 10
R_Combine bone 2655 OxA-39430-SUERC-86130
Phase grey clay with charcoals under Unio 12
R_Combine horse bone 2979 OxA-39363-SUERC-86136
R_Combine wooden pile OxA-39274/5-SUERC-86127
R_Combine deer pelvis OxA-39362- SUERC-86132
Phase vivip layer under clay in water
Sequence EXCAVATION 1
R_Date ND16 pit bone 1550 KIA-52978
R_Date bone 1677 KIA-52990
R_Date bone 1859 KIA-52997
Phase layer 17 intrusive
R_Combine bone 2493 OxA-39361-SUERC-86129
R_Date bone 1381 KIA-52980
Phase mis-stratified EN bones
Phase Rakushechny Yar EN terrestrial samples
Boundary Rakushechny Yar start
Sequence Rakushechny Yar 2016-19 trenches
7000
6500
6000
5500
5000
calibrated date (cal BC)

(previous page) **Supplementary Figure 5**: Chronological model of the early Neolithic occupation of Rakushechny Yar, with the same format as Supplementary Figure 1. Stratigraphic sequences from the 2016-19 excavations have been used to order the dates of samples. Duplicate 14C measurements on the same skeletal element or branch have been combined before calibration.

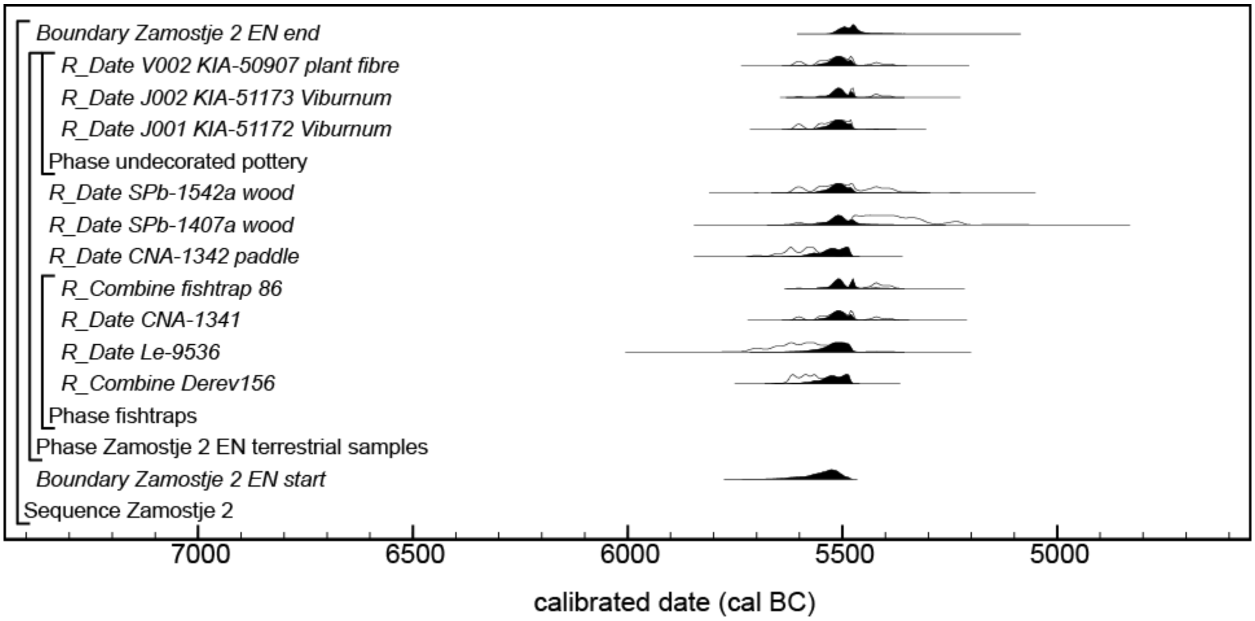

Supplementary Figure 6: Chronological model of the early Neolithic occupation of Zamostje 2, with the same format as Supplementary Figure 1. Duplicate 14C measurements on the same fish-trap have been combined before calibration.

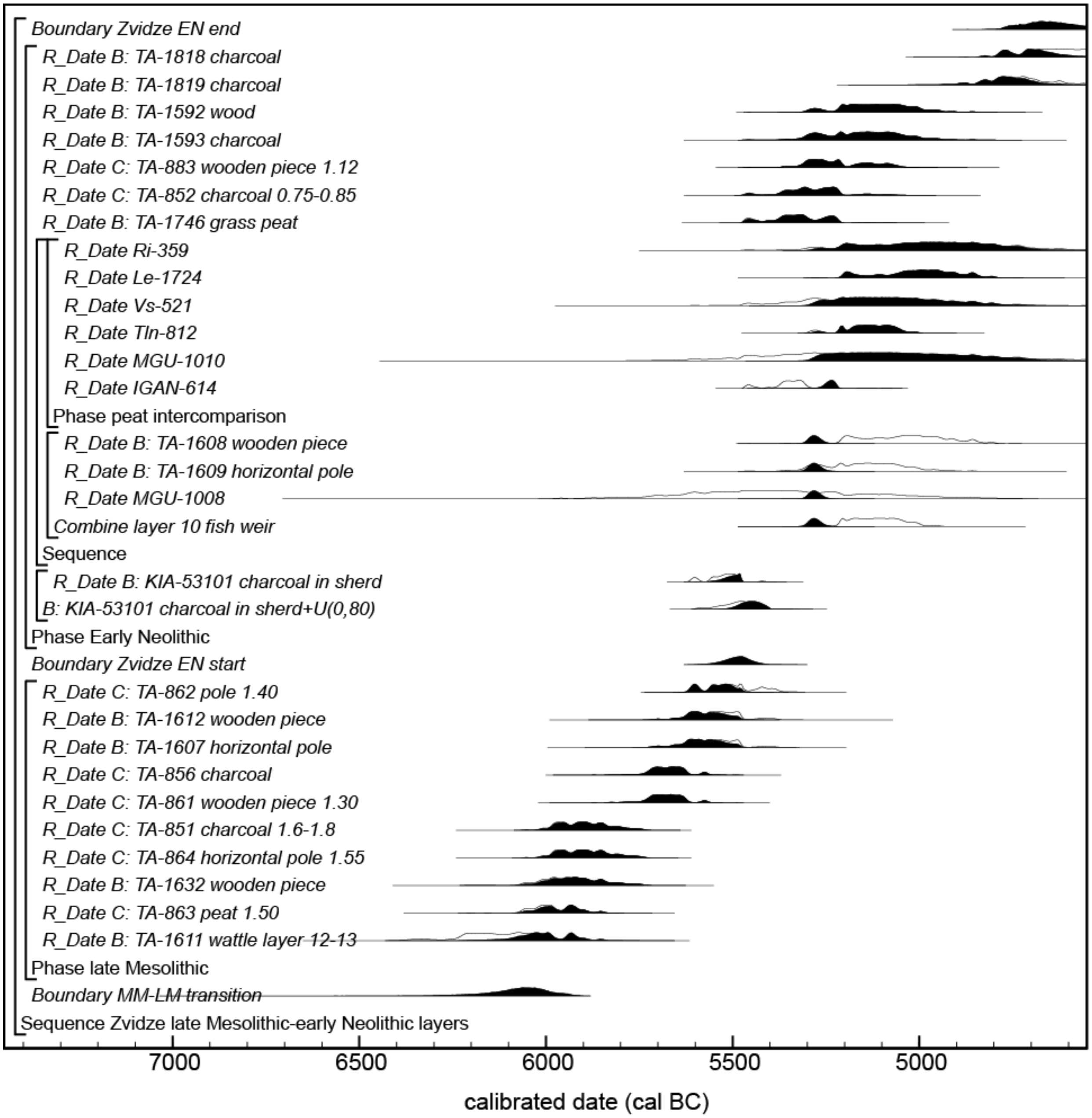

Supplementary Figure 7: Chronological model of the early Neolithic occupation of Zvidze, with the same format as Supplementary Figure 1. Duplicate 14C measurements on the same timber structure have been combined after calibration. The date of the charcoal inclusion in pottery, KIA-53101, has been shifted 0-80 years later to account for the intrinsic age of the wood.

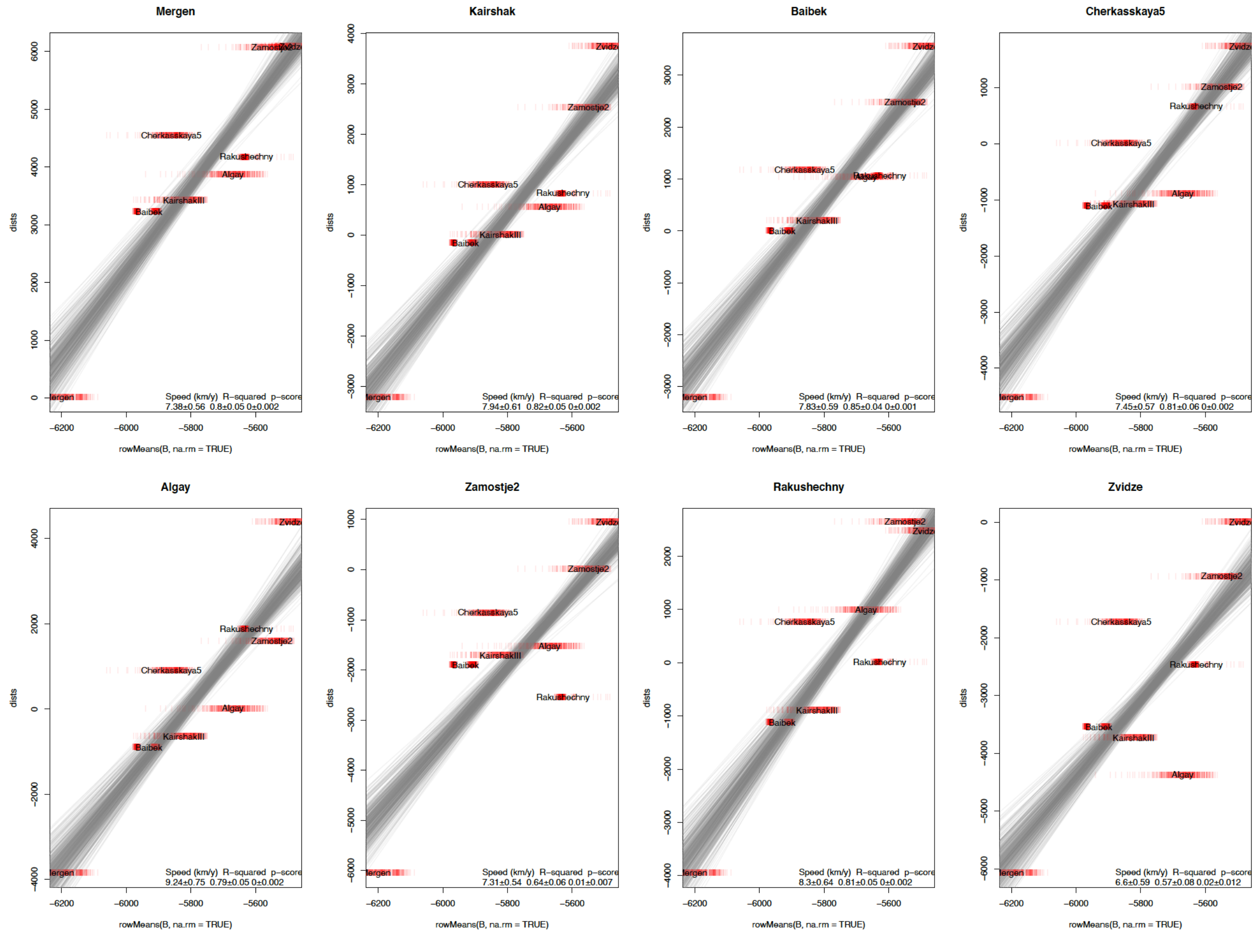

Supplementary Figure 8: Ordinary least-squares regression models of the diffusion rate of pottery including all sites

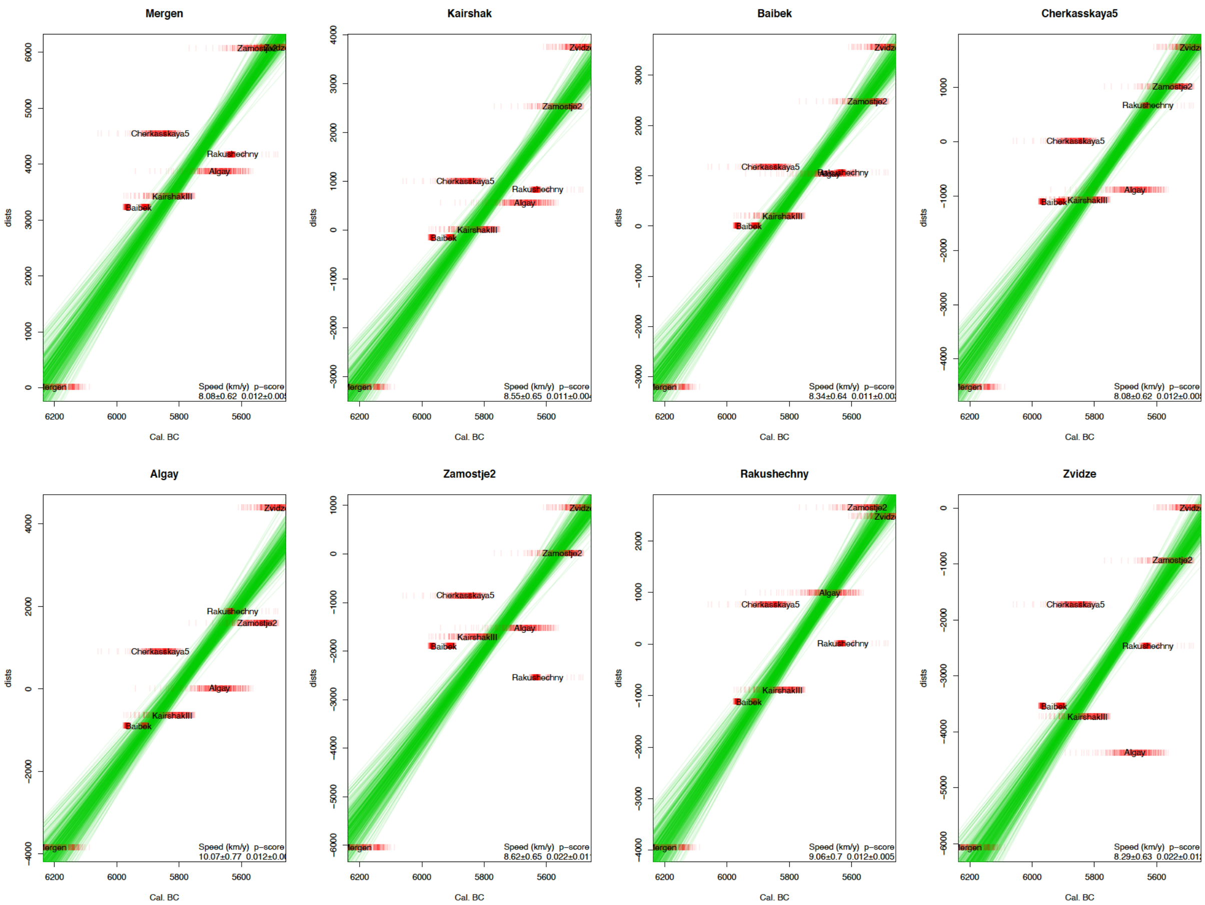

Supplementary Figure 9: Reduced major axis regression models of the diffusion rate of pottery

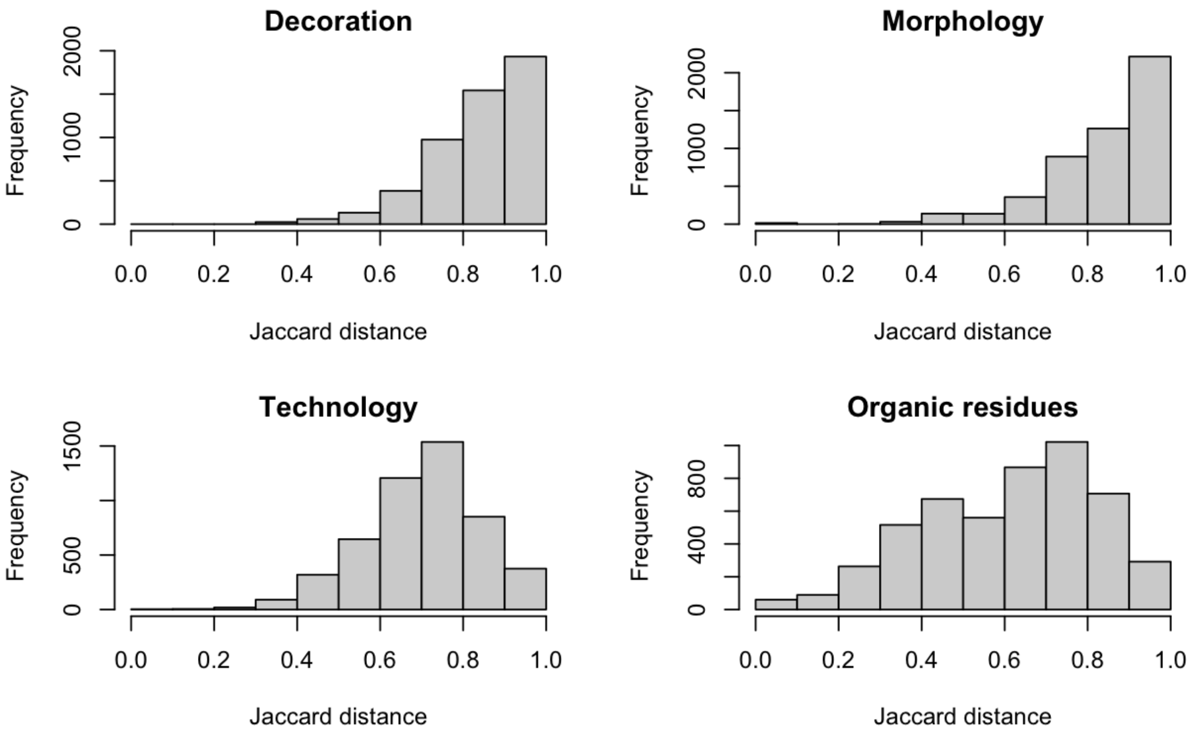

Supplementary Figure 10: Histograms illustrating the distribution of Jaccard distances

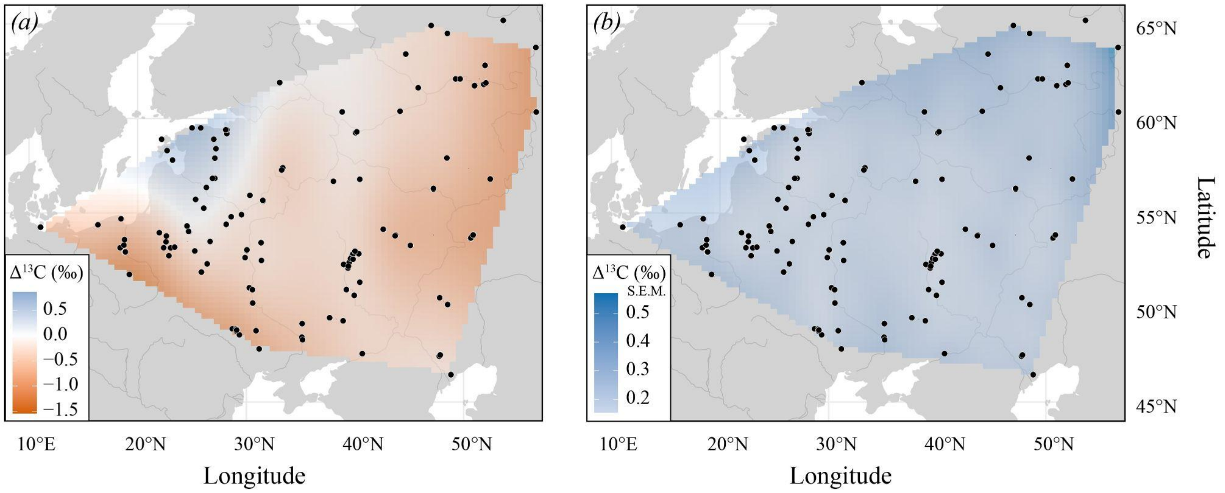

Supplementary Figure 12: (a) Output of AverageR model showing spatial estimates of $\Delta^{13}C$ ($C_{18:0}$-$C_{16:0}$) values of fatty acids . (b) Standard error of the mean for the estimated $\Delta^{13}C$ values shown in panel (a).

Supplementary Tables

Supplementary Table 1: Sites in the study

Site	Region	Country	Longitude	Latitude
Dubokray V	Dnepr-Dvina area	Russia	30.33	55.93
Rudnya Serteyskaya	Dnepr-Dvina area	Russia	31.53	55.65
Serteya X	Dnepr-Dvina area	Russia	31.53	55.63
Serteya XIV	Dnepr-Dvina area	Russia	31.51	55.67
Borisoglebskaya	Don region	Russia	42.05	51.35
Cherkasskaya	Don region	Russia	39.93	50.64
Cherkasskaya 3	Don region	Russia	39.93	50.64
Cherkasskaya 5	Don region	Russia	39.93	50.64
Dobroe 4	Don region	Russia	39.81	52.83
Dobroe 7	Don region	Russia	39.81	52.83
Dobroe 9	Don region	Russia	39.81	52.83
Dronikha	Don region	Russia	40.45	51.33
Karamyshevo 19	Don region	Russia	39.44	52.38
Karamyshevo 9	Don region	Russia	39.47	52.38
Kopanische	Don region	Russia	39.21	50.95
Ksizovo	Don region	Russia	38.96	52.28
Lipetskoe ozero	Don region	Russia	39.59	52.59
Monastyrskaya	Don region	Russia	40.44	51.34
mouth of Izlegoschi River	Don region	Russia	39.36	52.09
№380	Don region	Russia	39.74	52.76
Rybnoe ozero 2, site 202	Don region	Russia	39.81	52.56
Savitskoe	Don region	Russia	39.4	52.19
Staroe Torbeevo 11	Don region	Russia	40.35	52.84

Site	Region	Country	Longitude	Latitude
Vasilievsky kordon 3	Don region	Russia	39.98	52.98
Vasilievsky kordon 5	Don region	Russia	39.99	52.98
Vasilievsky kordon 7	Don region	Russia	40	52.98
Yarlukovskaya protoka	Don region	Russia	39.82	52.56
Hyrlo Biloi (Pidhorivka)	Donets region	Ukraine	38.91	49.3
Oleksandriia	Donets region	Ukraine	37.67	49.46
Akali	Eastern shore of the Baltic sea	Estonia	27.2	58.4
Ića	Eastern shore of the Baltic sea	Latvia	27.1	56.83
Jara 2	Eastern shore of the Baltic sea	Lithuania	25.32	55.72
Kääpa	Eastern shore of the Baltic sea	Estonia	27.1	57.9
Kalmaküla	Eastern shore of the Baltic sea	Estonia	27	58.9
Kõnnu	Eastern shore of the Baltic sea	Estonia	22.7	58.3
Kõpu I	Eastern shore of the Baltic sea	Estonia	22.2	58.9
Kretuonas	Eastern shore of the Baltic sea	Lithuania	26.08	55.25
Kroodi	Eastern shore of the Baltic sea	Estonia	25	59.5
Lommi III	Eastern shore of the Baltic sea	Estonia	28.2	59.2
Narva Joaorg	Eastern shore of the Baltic sea	Estonia	28.2	59.4
Osa	Eastern shore of the Baltic sea	Latvia	26.32	56.35
Riigiküla IV	Eastern shore of the Baltic sea	Estonia	28.1	59.4
Riigiküla VI	Eastern shore of the Baltic sea	Estonia	28.1	59.4
Ruhnu II	Eastern shore of the Baltic sea	Estonia	23.2	57.8
Vihasoo III	Eastern shore of the Baltic sea	Estonia	25.8	59.5
Zvidze	Eastern shore of the Baltic sea	Latvia	26.88	56.83
Kuromoila	Karelia	Russia	33.07	61.9
Rakucheshny Yar	Low Don	Russia	40.67	47.56
Algay	Low Volga	Russia	48.53	50.16
Varfolomeevka	Low Volga	Russia	47.8	50.52
Igren V	Lower Dnieper region	Ukraine	35.11	48.43

Site	Region	Country	Longitude	Latitude
Strilcha Skelia	Lower Dnieper region	Ukraine	35.17	48.29
Yosypivka-Lyman	Lower Dnieper region	Ukraine	35.13	49.15
Dudka	Masurian lake district	Poland	21.986	53.956
Szczepanki 8	Masurian lake district	Poland	21.986	53.956
Hryni	Middle Dnieper region	Ukraine	30.27	51.05
Romankiv 3	Middle Dnieper region	Ukraine	30.58	50.24
Strumil	Middle Dnieper region	Ukraine	30.56	50.94
Bolshaya Rakovka 2	Middle Volga	Russia	50.65	53.67
Chekalino IV	Middle Volga	Russia	50.91	53.86
Dubovskoe III	Middle Volga	Russia	47.2	56.27
Dubovskoe VIII	Middle Volga	Russia	47.22	56.25
Ilyinka	Middle Volga	Russia	50.64	53.65
Imerka 7	Middle Volga	Russia	42.62	54.12
Imerka 8	Middle Volga	Russia	42.59	54.14
Krasny gorodok	Middle Volga	Russia	50.64	53.65
Lebyzhinka 4	Middle Volga	Russia	50.67	53.68
Nizhnya Orlyanka II	Middle Volga	Russia	50.88	53.84
Otarskaya 6	Middle Volga	Russia	47.22	56.3
Ozimenki 2	Middle Volga	Russia	43.74	53.83
Podlesnoe III	Middle Volga	Russia	45.1	53.28
Potodeevo	Middle Volga	Russia	43.7	53.79
Asaviec 4	Middle Western Dvina River	Belarus	29.55	54.91
Biarešča 4	Middle Western Dvina River	Belarus	28.6	54.8
Zacennie	Middle Western Dvina River	Belarus	28.14	54.4
Brańsk, site 22	Neman river and Volhynia region	Poland	22.868	52.739
Drazdy 12	Neman river and Volhynia region	Belarus	26.66	53.49
Dubičiai 3	Neman river and Volhynia region	Lithuania	24.74	54.02
Glūkas 3	Neman river and Volhynia region	Lithuania	24.57	54.29

Site	Region	Country	Longitude	Latitude
Grądy-Woniecko, site 1	Neman river and Volhynia region	Poland	22.385	53.162
Gribaša 4	Neman river and Volhynia region	Lithuania	24.7	54.04
Jeroniki, site 2	Neman river and Volhynia region	Poland	23.023	53.154
Kamień 6	Neman river and Volhynia region	Belarus	26.39	52.31
Karaviškės 6	Neman river and Volhynia region	Lithuania	24.68	54.03
Krzemienne, site 2	Neman river and Volhynia region	Poland	23.398	53.194
Rusakova	Neman river and Volhynia region	Belarus	25.25	52.99
Senchytsi 3	Neman river and Volhynia region	Ukraine	25.86	51.87
Sośnia, site 1	Neman river and Volhynia region	Poland	22.601	53.476
Stacze, site 1	Neman river and Volhynia region	Poland	22.633	53.784
Varėnė 10	Neman river and Volhynia region	Lithuania	24.53	54.31
Baibek	Northern Caspian	Russia	48.84	46.45
Kairshak III	Northern Caspian	Russia	47.76	47.42
Kugat IV	Northern Caspian	Russia	47.85	47.49
Chernaya Vadia	Northern part of Eastern Europe	Russia	49.27	62.09
Chernoborskaya III	Northern part of Eastern Europe	Russia	53.65	65.19
Chudgudorjag	Northern part of Eastern Europe	Russia	51.04	61.75
Dutovo I	Northern part of Eastern Europe	Russia	56.65	63.75
En'ty IA	Northern part of Eastern Europe	Russia	51.01	61.73
Khomutovskoe boloto 2	Northern part of Eastern Europe	Russia	56.69	60.35
Kochmas	Northern part of Eastern Europe	Russia	49.68	62.09
Koneschelie	Northern part of Eastern Europe	Russia	47.02	64.92
Pezmog IV	Northern part of Eastern Europe	Russia	51.85	61.8
Prilukskaya	Northern part of Eastern Europe	Russia	45.82	61.62
Ugdym IA	Northern part of Eastern Europe	Russia	52.05	61.88
Ursinka 1	Northern part of Eastern Europe	Russia	56.69	60.34
Vis I	Northern part of Eastern Europe	Russia	51.98	62.81
Vis II	Northern part of Eastern Europe	Russia	51.98	62.81

Site	Region	Country	Longitude	Latitude
Yavron'ga 1	Northern part of Eastern Europe	Russia	44.68	63.41
Zubovo	Northern part of Eastern Europe	Russia	48.5	64.5
Kochurovskoe I	Perm	Russia	52.45	56.8
Koshkinskaya	Perm	Russia	48.46	57.91
Bazkiv Ostriv	Southern Bug region	Ukraine	29.36	48.55
Dobrianka 1	Southern Bug region	Ukraine	30.89	48.77
Dobrianka 3	Southern Bug region	Ukraine	30.89	48.77
Hlynske I	Southern Bug region	Ukraine	29.09	48.74
Pechera I	Southern Bug region	Ukraine	28.71	48.87
Puhach II	Southern Bug region	Ukraine	31.18	47.81
Samchyntsi I	Southern Bug region	Ukraine	29.05	48.87
Shchurivtci-Shymanovske II	Southern Bug region	Ukraine	29.11	48.82
Shchurivtsi-Porih	Southern Bug region	Ukraine	29.14	48.83
Skybyntsi	Southern Bug region	Ukraine	29.36	48.56
Sokiltsi I	Southern Bug region	Ukraine	29.11	48.8
Sokiltsi II	Southern Bug region	Ukraine	29.11	48.8
Sokiltsi VI	Southern Bug region	Ukraine	29.11	48.81
Dąbki, site 9	Southern shore of the Baltic sea	Poland	16.33	54.38
Grube-Rosenhof LA 58	Southern shore of the Baltic sea	Germany	11.05	54.25
Dubovy Loh 5	Upper Dniepr	Belarus	31.38	52.49
Lučyn Barok	Upper Dniepr	Belarus	30.03	53
Lučyn Barok Siamionaŭski	Upper Dniepr	Belarus	30.05	53.02
Prorva 2	Upper Dniepr	Belarus	30.05	53.05
Taŭstyki 1	Upper Dniepr	Belarus	29.86	52.62
Turja	Upper Dniepr	Belarus	31.35	53.43
Vasilievičy 3	Upper Dniepr	Belarus	29.88	52.64
Sakhtysh 2	Upper Volga	Russia	40.45	56.78
Sakhtysh 2a	Upper Volga	Russia	40.45	56.78

Site	Region	Country	Longitude	Latitude
Sakhtysh 8	Upper Volga	Russia	40.44	56.78
Zamostje 2	Upper Volga	Russia	38.01	56.68
Kochische II	Valday	Russia	33.24	57.29
Schepochnik	Valday	Russia	33.35	57.39
Zalesie	Valday	Russia	33.25	57.27
Dzikowo, site 26	Vistula river	Poland	18.791	52.936
Kałdus, site 3	Vistula river	Poland	18.382	53.161
Lutomiersk-Wrząca	Vistula river	Poland	19.232	51.751
Mgoszcz, site 2	Vistula river	Poland	18.723	53.301
Osiek, site 9	Vistula river	Poland	18.815	52.942
Rzucewo 1	Vistula river	Poland	18.455	54.694
Sąsieczno, site 3 (concentration 1)	Vistula river	Poland	18.859	52.942
Sąsieczno, site 4	Vistula river	Poland	18.86	52.941
Sąsieczno, site 4 (concentration 1)	Vistula river	Poland	18.86	52.941
Wełcz Wielki, site 10A	Vistula river	Poland	18.781	53.591
Wełcz Wielki, site 10B	Vistula river	Poland	18.782	53.589
Berezovaya Slobodka	Vologda region	Russia	44.15	60.38
Karavaikha 4	Vologda region	Russia	38.82	60.35
Liminskaya	Vologda region	Russia	40.05	59.24
Veksa I	Vologda region	Russia	40.18	59.29
Veksa III	Vologda region	Russia	40.17	59.29

Supplementary Table 2: **New and/or previously unpublished ^{14}C results**

Site	Lab. code	^{14}C age BP	^{14}C error	$\delta^{13}C$ ‰	$\delta^{15}N$‰	C %	N %	C/N ratio	Sample material	Archaeological excavation and contextual details
Algay	SUERC-88034	6638	26	-19.2	7.6			3.4	koulan bone (6.4% collagen yield)	square 18-22; layer 30
Algay	SUERC-88039	6669	26	-19.4	8.7			3.3	auroch bone (8.6% collagen yield)	square 18-22; layer 30
Algay	SUERC-88038	6623	26	-16.0	9.5			3.3	saiga bone (13.9% collagen yield)	square 18-22; layer 31
Algay	SUERC-88040	6380	27	-17.6	10.3			3.1	saiga bone (5.1% collagen yield)	square 23-25 28-30; layer 31
Algay	SUERC-88041	6239	23	-16.2	8.5			3.2	indet. bone (6% collagen yield)	square 23-25 28-30; layer 31
Algay	SPb-3346	6882	100						bone	
Algay	SPb-3348	6205	120						bone	
Algay	SPb-3360	6200	70						bone	
Baibek	DeA-20722	6992	37						bone	2013 exc. 1 (ashpit 2)
Baibek	DeA-20723	6976	37						bone	2013 exc. 1 (ashpit 2)
Baibek	DeA-20724	7097	41						bone	2013 exc. 1 (pit 1)
Baibek	DeA-20725	7034	43						bone	2013 exc. 1 (pit 1)

Site	Lab. code	^{14}C age BP	^{14}C error	$\delta^{13}C$ ‰	$\delta^{15}N$‰	C %	N %	C/N ratio	Sample material	Archaeological excavation and contextual details
Baibek	OxA-39162	7010	27	-19.5	8.7	45.0		3.2	bone (36.26% collagen yield)	2013 exc.1 sq.8; cultural layer 1
Baibek	DeA-20726	7056	38						bone	2014 E part sq.393; layer 4 (pit 1)
Baibek	DeA-20727	7036	37						bone	2014 E part sq.393; layer 4 (pit 1)
Baibek	DeA-20728	6998	39						bone	2014 exc.1 E part sq.374 (ashpit 3)
Baibek	DeA-20729	7023	39						bone	2014 exc.1 situation 3 sq.135 (pit 4)
Baibek	DeA-20957	6952	40						red deer bone	2014 sq.393 397 (ash-pit 6)
Baibek	DeA-20958	7012	34						koulan bone	2014 sq.393 397 (ash-pit 6)
Baibek	DeA-20730	7023	39						koulan bone	2017 (pit 1)
Baibek	OxA-39163	7060	28	-19.4	10.5	44.1		3.2	bone (19.39% collagen yield)	2017 exc.1 (pit 1 dwelling 1)
Baibek	OxA-39164	7030	28	-20.1	8.8	44.1		3.2	bone (8.5% collagen yield)	2017 exc.1 E-W section sq. 443; low level
Baibek	OxA-39165	7012	28	-19.1	9.3	44.2		3.2	bone (12.62% collagen yield)	2017 exc.1 E-W section sq. 443; low level
Baibek	OxA-39166	7035	27	-19.7	8.5	44.0		3.2	bone (18.84% collagen yield)	2017 exc.1 N part N-S section sq. 437; low level
Baibek	OxA-39296	7030	27	-19.4	9.3			3.2	bone (7.3% collagen yield)	2017 exc.1 N part sq. 422; level 14 (dwelling)
Baibek	OxA-39074	7048	25	-19.5	7.5	45.9		3.2	bone	2017 exc.1 N part sq. 427; level8 (dwelling)

Site	Lab. code	^{14}C age BP	^{14}C error	δ^{13}C ‰	δ^{15}N‰	C %	N %	C/N ratio	Sample material	Archaeological excavation and contextual details
Baibek	OxA-39332	6989	32	-19.6	7.7			3.4	bone (<1% collagen yield)	2017 exc.1 N part sq. 429; level 3
Baibek	OxA-39075	7023	24	-17.2	12.0	46.6		3.2	saiga bone	2017 exc.1 sq. 416; layer 6
Baibek	OxA-39076	7016	24	-19.2	11.4	46.1		3.2	koulan bone	2017 excavation 1 northern part sq. 460; level3 (ashpit 7)
Baibek	OxA-39077	7024	25	-19.8	8.2	45.6		3.2	red deer bone	2017 fire-place; layer 12
Baibek	OxA-39133	6994	29	-19.2	8.5	40.8		3.2	bone (15.55% collagen yield)	2017 N part N-S section sq. 437; low level (low bottom part of the dwelling)
Baibek	OxA-39232	6978	28	-17.8	9.8	42.0		3.2	koulan bone (17.1% collagen yield)	2017 northern part; level 2 (pit 4)
Baibek	OxA-39134	6994	28	-19.3	9.1	43.0		3.2	red deer bone (18.3% coll. yield)	2017 pit 4; level 2
Baibek	OxA-39518	7041	27	-19.4	7.3	42.1		3.2	red deer bone (4.4% collagen yield)	2017 sq. 424; layer 6
Baibek	OxA-39136	6999	29	-19.0	8.5	41.7		3.2	saiga bone (15.9% collagen yield)	2017 sq. 428 429 435 436; layer 12 (fire-place)
Baibek	OxA-39137	7020	28	-18.6	8.9	43.0		3.2	koulan bone (47.4% collagen yield)	2018 eastern part square 543; level 3
Baibek	OxA-39138	7002	29	-16.3	14.3	42.8		3.2	saiga bone (15.85% collagen yield)	2018 eastern part square 543; level 3

Site	Lab. code	^{14}C age BP	^{14}C error	$\delta^{13}C$ ‰	$\delta^{15}N$‰	C %	N %	C/N ratio	Sample material	Archaeological excavation and contextual details
Baibek	OxA-39139	7022	29	-18.7	10.5	42.4		3.2	red deer bone (10.4% collagen yield)	2018 eastern part square 543; level 3
Baibek	DeA-20731	7037	37						koulan bone	2018; layer 12 (fire-place)
Baibek	DeA-20732	7026	76						koulan bone	2018; level 2 (ash-pit 13)
Kairshak III	OxA-40228	6908	26	-20.3	6.6	45.0		3.2	koulan bone (6.6% collagen yield)	sample 1
Kairshak III	SUERC-93642	6973	44	-19.1	10.2			3.2	koulan bone (9.1% collagen yield)	sample 2
Kairshak III	OxA-40229	6890	27	-19.8	7.2	42.6		3.2	koulan bone (1.1% collagen yield)	sample 3
Kairshak III	SUERC-100998	6934	27	-20.4	8.1	33.1	11.6	3.3	bone (6.9% collagen yield)	sq.104 pit; level 5 (shovel depth)
Kairshak III	SUERC-100999	6885	25	-19.4	9.8	36.3	12.4	3.4	bone (9.6% collagen yield)	sq.105; level 4 (shovel depth)
Kairshak III	SUERC-101001	6872	25	-19.0	10.9	33.8	12.3	3.2	bone (6% collagen yield)	sq.32; lower level
Kairshak III	SUERC-101000	6901	27	-20.2	8.0	36.3	12.7	3.3	bone (7.6% collagen yield)	sq.99 pit; level 5 (shovel depth)
Rakushechny Yar	SUERC-100994	6450	26	-20.6	6.5	41.2	14.1	3.4	bone (6.4% collagen yield)	Belanovskaya exc. 2; layer 11

Site	Lab. code	^{14}C age BP	^{14}C error	δ^{13}C ‰	δ^{15}N‰	C %	N %	C/N ratio	Sample material	Archaeological excavation and contextual details
Rakushechny Yar	SUERC-86130	6658	28	-19.9	6.6			3.2	deer bone (9.5% collagen yield)	excavation 1; gray clay with charcoals under Unio 12
Rakushechny Yar	OxA-39431	6681	24	-20.5	7.3	44.2		3.2	roe deer bone (5.9% collagen yield)	excavation 1; gray layer (fewer shells more large fish and pottery)
Rakushechny Yar	SUERC-86131	6684	28	-20.2	7.6			3.2	roe deer bone (10.2% collagen yield)	excavation 1; gray layer (fewer shells more large fish and pottery)
Rakushechny Yar	OxA-39359	6671	27	-18.6	6.6			3.2	cow bone	excavation 1; light gray sand with shells (probably layer 18 earlier than layer 17)
Rakushechny Yar	OxA-39361	6695	26	-20.3	9.4			3.2	deer bone (5% collagen yield)	excavation 1; Unio 10
Rakushechny Yar	SUERC-86129	6766	28	-20.1	9.9			3.2	deer bone (12.4% collagen yield)	excavation 1; Unio 10
Rakushechny Yar	OxA-39362	6656	26	-20.4	7.0			3.2	pig bone (3.1% collagen yield)	excavation 1; vivip layer under clay in water
Rakushechny Yar	OxA-39363	6651	25	-20.8	5.3			3.2	deer bone (8% collagen yield)	excavation 1; vivip layer under clay in water
Rakushechny Yar	SUERC-86132	6638	28	-20.4	7.7			3.2	pig bone (4.5% collagen yield)	excavation 1; vivip layer under clay in water
Rakushechny Yar	SUERC-86136	6677	28	-20.3	5.9			3.1	deer bone (11.2% collagen yield)	excavation 1; vivip layer under clay in water

Site	Lab. code	^{14}C age BP	^{14}C error	$\delta^{13}C$ ‰	$\delta^{15}N$‰	C %	N %	C/N ratio	Sample material	Archaeological excavation and contextual details
Rakushechny Yar	OxA-39360	6661	26	-20.5	8.9			3.2	pig bone (2.5% collagen yield)	excavation 4; Unio 2
Rakushechny Yar	SUERC-86128	6676	28	-20.5	9.1			3.3	pig bone (2.6% collagen yield)	excavation 4; Unio 2
Rakushechny Yar	OxA-39430	6680	24	-20.1	6.3	43.6		3.2	deer bone (6% collagen yield)	excavation; gray clay with charcoals under Unio 12
Rakushechny Yar	KIA-52980	6746	27						pig bone (0.096 collagen yield)	ND16; layer 15a (upper part square A7)
Rakushechny Yar	KIA-52990	6565	28						indet. bone (0.031 collagen yield)	ND16; layer 17 (lower part)
Rakushechny Yar	KIA-52991	6708	28	-19.8	5.2	39.8	15.0	3.1	sheep bone (0.027 collagen yield)	ND16; layer 17 (Unio 6)
Rakushechny Yar	KIA-52996	6596	29	-20.0	6.0	40.8	15.2	3.1	medium mammal bone (0.136 collagen yield)	ND16; layer 17 (Unio shell #2)
Rakushechny Yar	KIA-52997	6579	30	-21.0	5.9	41.6	15.3	3.2	large mammal bone (0.044 collagen yield)	ND16; layer 17 (Unio shell #2)
Rakushechny Yar	KIA-52978	6702	28						large mammal bone (0.065 collagen yield)	ND16; pit (lowest Unio layer)
Rakushechny Yar	OxA-39364	6630	26	-21.2	6.7			3.2	roe deer bone (2.3% collagen yield)	trench layer 5a vivip
Rakushechny Yar	SUERC-86137	6355	28	-21.1	5.5			3.2	horse bone (2.4% collagen yield)	trench layer 5a vivip

Site	Lab. code	^{14}C age BP	^{14}C error	δ^{13}C ‰	δ^{15}N‰	C %	N %	C/N ratio	Sample material	Archaeological excavation and contextual details
Rakushechny Yar	SUERC-86138	6579	28	-21.0	6.6			3.2	roe deer bone (3% collagen yield)	trench layer 5a vivip
Rakushechny Yar	OxA-39274	6652	23	-24.2					wood	vivip layer under clay in water
Rakushechny Yar	SUERC-86127	6649	28	-25.1					wood	vivip layer under clay in water
Zamostje 2	KIA-51172	6566	36						*Viburnum* berry	potsherd J001
Zamostje 2	KIA-51173	6527	32						*Viburnum* berry	potsherd J002

Supplementary Table 3: previously-published ^{14}C ages used in chronological models

Site	Laboratory code	^{14}C age BP	^{14}C error	δ^{13}C ‰	δ^{15}N‰	Material	Ref.
Algay	AAR-21892	6318	33			bone	18
Algay	AAR-21893	6605	32			charcoal	18
Algay	Poz-76004	6490	40			charcoal	18
Algay	SPb-1411	6360	250			charcoal	18
Algay	SPb-1477	6479	70			bone	18
Algay	SPb-1478	6577	80			bone	18
Algay	SPb-1509	6654	80			bone	18
Algay	SPb-1510	6820	80			bone	18
Algay	SPb-2038	6284	100			bone	18
Algay	UCIAMS-229409	6440	25			bone	19
Baibek	SPb-1444	6868	80			burnt deposit	20
Baibek	SPb-1708	6868	70			bone	20
Baibek	SPb-1709	6955	80			bone	20
Baibek	SPb-1711	7099	100			bone	20
Baibek	SPb-1712	6827	100			charcoal	20
Baibek	SPb-1713	6948	120			charcoal	20
Baibek	SPb-1715	7041	120			charcoal	20
Baibek	SPb-1721	6952	80			bone	20
Baibek	SPb-1722	6849	100			sediment	20
Baibek	SPb-2785	6935	75			bone	5
Baibek	SPb-2786	6925	70			bone	5
Baibek	SPb-2787	6950	70			bone	5
Baibek	SPb-2789	6936	80			bone	5
Baibek	SPb-973	6955	80			bone	20
Baibek	Ua-50260	6986	44			charcoal	20
Cherkasskaya 5	OxA-39520	6999	27	-21.0	3.2	bone	10

Site	Laboratory code	^{14}C age BP	^{14}C error	δ^{13}C ‰	δ^{15}N‰	Material	Ref.
Cherkasskaya 5	OxA-39521	7130	26	-20.7	3.1	tooth	10
Cherkasskaya 5	OxA-39522	6982	26	-20.9	3.6	bone	10
Cherkasskaya 5	SUERC-86147	6987	28	-29.9		plant macrofossil	10
Cherkasskaya 5	SUERC-86148	6966	28	-20.8	3.0	bone	10
Cherkasskaya 5	SUERC-86149	6943	28	-21.0	2.9	bone	10
Cherkasskaya 5	SUERC-86150	6950	28	-20.8	5.8	bone	10
Cherkasskaya 5	SUERC-86151	7140	28	-20.8	3.3	tooth	10
Cherkasskaya 5	SUERC-86156	6938	28	-20.9	3.3	bone	10
Cherkasskaya 5	SUERC-86157	6908	28	-21.1	2.3	tooth	10
Cherkasskaya 5	SUERC-86158	6886	28	-22.0	6.4	bone	10
Kairshak III	Hela-4165	6996	36			bone	20
Kairshak III	Ki-14633	7190	80			bone	20
Kairshak III	Ki-14634	7010	80			bone	20
Kairshak III	SPb-316	7030	100			bone	20
Rakushechny Yar	DeA-20969	6634	34	-20.1	5.9	bone	11
Rakushechny Yar	DeA-20970	6568	33	-20.4	4.6	bone	11
Rakushechny Yar	DeA-20971	6584	33	-20.3	4.5	bone	11
Rakushechny Yar	DeA-20972	6462	33	-19.9	4.8	bone	11
Rakushechny Yar	DeA-21601	4535	35			bone	11
Rakushechny Yar	KIA-52981	6590	28			bone	21
Rakushechny Yar	KIA-52982	6649	27	-19.8	5.9	bone	21
Rakushechny Yar	KIA-52983	6626	28	-20.2	5.1	bone	21
Rakushechny Yar	KIA-52984	6655	28	-20.0	5.1	bone	21
Rakushechny Yar	KIA-52985	6632	28	-19.2	6.5	bone	21
Rakushechny Yar	KIA-52986	6683	29	-19.9	6.2	bone	21
Rakushechny Yar	KIA-52987	6681	28	-20.7	7.9	bone	21
Rakushechny Yar	KIA-52988	6645	27	-19.6	6.0	bone	21
Rakushechny Yar	KIA-52989	6711	27	-19.9	5.4	bone	21
Rakushechny Yar	KIA-52992	6652	28	-19.6	5.4	bone	21
Rakushechny Yar	KIA-52993	6650	29	-19.5	5.4	bone	21

Site	Laboratory code	^{14}C age BP	^{14}C error	δ^{13}C ‰	δ^{15}N‰	Material	Ref.
Rakushechny Yar	KIA-52994	6643	28	-20.1	5.5	bone	[21]
Rakushechny Yar	KIA-52995	6666	30	-20.5	7.3	bone	[21]
Rakushechny Yar	OxA-39619	6604	26	-19.9	6.7	bone	[11]
Rakushechny Yar	SUERC-86126	4179	28	-18.5	11.5	bone	[11]
Rakushechny Yar	SUERC-88042	2128	25	-20.3	9.9	bone	[11]
Rakushechny Yar	SUERC-88043	6644	27	-19.7	5.0	bone	[11]
Rakushechny Yar	SUERC-94517	1855	31	-19.6	7.4	tooth	[11]
Rakushechny Yar	SUERC-94518	5433	31	-18.9	9.1	bone	[11]
Zamostje 2	Beta-283033	6550	40			wood (worked)	[22]
Zamostje 2	CNA-1081	6452	43			wood (worked)	[22]
Zamostje 2	CNA-1341	6539	43			wood (worked)	[22]
Zamostje 2	CNA-1342	6676	47			wood (worked)	[22]
Zamostje 2	CNA-1345	6646	39			wood (worked)	[22]
Zamostje 2	KIA-50907	6545	48			plant macrofossil	[13]
Zamostje 2	Le-9523	6730	150			wood (worked)	[22]
Zamostje 2	Le-9536	6670	80			wood (worked)	[22]
Zamostje 2	SPb-1407	6450	90			wood from gyttja	[22]
Zamostje 2	SPb-1542	6539	70			wood from gyttja	[22]
Zvidze	IGAN-614	6360	40			peat	[17]
Zvidze	KIA-53101	6567	33	-26.8		charcoal	[14]
Zvidze	Le-1724	6080	70			peat	[17]
Zvidze	MGU-1008	6450	250			wood	[17]
Zvidze	MGU-1010	6200	240			peat	[17]
Zvidze	Ri-359	6050	150			peat	[17]
Zvidze	TA-1592	6170	70			wood	[17]
Zvidze	TA-1593	6210	80			charcoal	[17]
Zvidze	TA-1607	6630	80			wood	[17]
Zvidze	TA-1608	6110	80			wood	[17]
Zvidze	TA-1609	6210	80			wood	[17]
Zvidze	TA-1611	7240	100			wood	[17]

Site	Laboratory code	^{14}C age BP	^{14}C error	δ^{13}C ‰	δ^{15}N‰	Material	Ref.
Zvidze	TA-1612	6610	80			wood	17
Zvidze	TA-1632	7060	80			wood	17
Zvidze	TA-1746	6350	60			peat	17
Zvidze	TA-1818	5770	60			charcoal	17
Zvidze	TA-1819	5870	60			charcoal	17
Zvidze	TA-851	7020	60			charcoal	17
Zvidze	TA-852	6315	60			charcoal	17
Zvidze	TA-856	6770	60			charcoal	17
Zvidze	TA-861	6780	60			wood	17
Zvidze	TA-862	6535	60			wood	17
Zvidze	TA-863	7110	60			peat	17
Zvidze	TA-864	7020	60			wood	17
Zvidze	TA-883	6260	60			wood	17
Zvidze	Tln-812	6195	40			peat	17
Zvidze	Vs-521	6180	150			peat	17

Supplementary Table 4: Estimated average rates of transmission

Site	OLS			RMA	
	Speed (km yr^{-1})	R-squared	p-score	Speed (km yr^{-1})	p-score
Mergen	7.38±0.56	0.8±0.05	0.004±0.002	8.08±0.62	0.012±0.005
Kairshak	7.94±0.61	0.82±0.05	0.004±0.002	8.55±0.65	0.011±0.004
Baibek	7.83±0.59	0.85±0.04	0.002±0.001	8.34±0.64	0.011±0.003
Cherhasskaya 5	7.45±0.57	0.81±0.06	0.004±0.002	8.08±0.62	0.012±0.005
Algay	9.24±0.75	0.79±0.05	0.004±0.002	10.07±0.77	0.012±0.004
Zamostje	7.31±0.54	0.64±0.06	0.01±0.007	8.62±0.65	0.022±0.011
Rakushechny Yar	8.3±0.64	0.81±0.05	0.004±0.002	9.06±0.7	0.012±0.005
Zvidze	6.6±0.59	0.57±0.08	0.02±0.012	8.29±0.63	0.022±0.012

Supplementary Table 5: Modelled front speeds in archaeological case studies

Case study	Front speed (km yr^{-1})	Reference
Spread of pottery from Asia	1.25 (corridors)<br>0.25 (elsewhere)	[3]
Spread of pottery from Africa	3.23 (corridors)<br>0.46 (elsewhere)	[3]
Neolithic culture in Europe	0.6 to 1.3	[26]
Neolithic LBK expansion	0.8±0.6	[27]
Neolithic Western Cardial expansion	3.3±3.7 (Mediterranean)<br>1.6±1.2 (temperate)	[27]
Neolithic expansion in Western Mediterranean	> 5	[28]
Neolithic painted pottery expansion	0 to 1.7	[29]
Neolithic LBK expansion	0 to 1.7	[29]
Neolithic Funnelbeaker expansion	0 to 1.2	[29]
Neolithic expansion in Scandinavia	0.44 to 0.84	[30]
Bantu agriculture in Africa	0.03 to 4.3, depending on biome	[31]
Spread of pastoral economy in southern Africa	1.4 to 2.8	[32]
Spread of HG pottery in Eastern Europe	6.6±0.59 to 10.07±0.77	This study

Supplementary Table 6: Pottery traits

Table	Description	Code
Morphology	Sherds from rim	R
Morphology	Sherds from belly	Be

Table	Description	Code
Morphology	Sherds from belly-rib	Ber
Morphology	Sherds from low part	lp
Morphology	Sherds from bottom	B
Morphology	Sherds from whole vessel	w
Morphology	Rim rim-flat	Rf
Morphology	Rim pointed	Rp
Morphology	Rim pointed-flattened	Rpf
Morphology	Rim roundish	Rr
Morphology	Rim pointed-oblique inside	Ro
Morphology	Rim thickened	Rt
Morphology	Rim oblique outside	Roo
Morphology	Rim inturned(c)	Ri
Morphology	Rim outurned	R0.5
Morphology	Rim thickened wall	R0.6
Morphology	Rim angle	A
Morphology	Diameter 5 cm	D5
Morphology	Diameter 10-15 cm	D10
Morphology	Diameter 16-19 cm	D16
Morphology	Diameter 20-25 cm	D20
Morphology	Diameter more 25 cm	D30
Morphology	Form Form-C (globe)	Fc
Morphology	Form demi-globe-bowl	Fb
Morphology	Form ellipse closed	Fec
Morphology	Form ellipse open	Feo
Morphology	Form lamp (ovoid)	Fl

Table	Description	Code
Morphology	Form ladle	Fla
Morphology	Form S-form(cone+globe)	Fs
Morphology	Form S+cones (Ert)	Fsc
Morphology	Form S slightly	Fss
Morphology	Form cut oval+globe (Lys Gora type)	FLG
Morphology	Form critical point	cr.p.
Morphology	Form vertical-cylinder	Fv
Morphology	Form neck+ellipse/globe	Fn
Morphology	Form closed (cone-ellipse-	Fc.1
Morphology	Form biconical	Fbi
Morphology	Form cylinder-cone-angles-low	X0.9
Morphology	Volume 0.5 l	Vs
Morphology	Volume 1-2 l	Vs1
Morphology	Volume 3-5 l	Vm
Morphology	Volume 6-10 l	Vm1
Morphology	Volume 11-20 l	Vb
Morphology	flat base	X0.10
Morphology	diameter	Bf
Morphology	flat base - rounded edges	D
Morphology	flat with an edge	Bfed
Morphology	Angle	Dfe
Morphology	roundish	A.1
Morphology	r. flattened	Br
Morphology	r.with small lump in extremity	Brf
Morphology	conical	Brc

Table	Description	Code
Morphology	c. thickened	Bc
Morphology	c.flattened	Bct
Morphology	Angle	Bcf
Morphology	repair holes	Urh
Morphology	fc out	Ufco
Morphology	fc in	Ufci
Morphology	soot	Uso
Morphology	splits	Ud
Morphology	abrasion	Ue
Technology	Paste/Temper dense paste	Td
Technology	Paste/Temper shell	Tsh
Technology	Paste/Temper sand medium-coarse grain	Ts
Technology	Paste/Temper thin sandy paste	Tsandt
Technology	Paste/Temper hematite	Th
Technology	Paste/Temper crushed stones	Tcrs
Technology	Paste/Temper mica	Tm
Technology	Paste/Temper long org remains	Tlo
Technology	Paste/Temper org-roundish	Tor
Technology	Paste/Temper chamotte	Tch
Technology	Paste/Temper size-big/medium	cbm
Technology	Paste/Temper size-small/medium	csm
Technology	Abundant	a
Technology	Moderate	m
Technology	Rare	s
Technology	modelling coils N (up to 1-2 cm)	McN

Table	Description	Code
Technology	modelling N elongated (up to 3 cm)	McNe
Technology	modelling N inverse	McNi
Technology	modelling S-slabs	Mss
Technology	modelling N parallel-slabs	MNs
Technology	modelling slabs combined - two layers	MNh
Technology	modelling slabs- U junction	Msu
Technology	modelling U	MU
Technology	modelling H	MH
Technology	modelling beating	Mb
Technology	thickness 0.4-0.6	Th5
Technology	thickness 0.7-0.8	Th7
Technology	thickness 0.9-1.2	Th1
Technology	thickness 1.3-1.8	Th2
Technology	Surface treatment smooth in	Ssi
Technology	Surface treatment smooth out	Sso
Technology	Surface treatment scratches inner	Sscri
Technology	Surface treatment scratches out	Sscr0
Technology	Surface treatment polishing out	Spo
Technology	Surface treatment polishing in	Spi
Technology	Surface treatment single linear traces	Sl
Technology	Surface treatment smoothed scratches	Sss
Technology	Surface treatment brushing in	Sb
Technology	Surface treatment ochre	So
Technology	Surface treatment polished with ochre	Spo
Technology	Surface treatment thin clay layer out	Scl

Table	Description	Code
Decoration	Impressions graphical sign - roundish impressions by bird bone?	Gr
Decoration	Impressions dots	Gd
Decoration	Impressions round impr.	Gri
Decoration	Impressions oval	Go
Decoration	Impressions triangular	Gt
Decoration	Impressions stick-edge made-UV	Gse
Decoration	Impressions denticulated big round	Gdb
Decoration	Impressions denticulated small round	Gds
Decoration	Impressions notches by nail	Gn
Decoration	Impressions incision hor	Gi
Decoration	Impressions incision vert	Giv
Decoration	Impressions denticulated vertical	Gde
Decoration	Impressions quadrangular by stick	Gq
Decoration	Impressions quadrangular twinned	Gqt
Decoration	Impressions scratches group - comb traced	Gs
Decoration	Impressions scratches-all	Gsa
Decoration	Impressions line	Gl
Decoration	Impressions short line	Gsl
Decoration	Impressions two-teeth stamp	Gtt
Decoration	Impressions short comb forest style	Gcs
Decoration	Impressions short comb 'impresso"	Gsc
Decoration	Impressions oval-comb	Goc
Decoration	Impressions short comb-retreated	Gscr
Decoration	Impressions long comb	Gcl
Decoration	Impressions fish bone stamp	Gfb

Table	Description	Code
Decoration	Impressions oval left by extremity of a comb stamp	Gol
Decoration	Impressions false cord impr	Gfc
Decoration	Impressions tuck (Защип)	Gt
Decoration	Motifs +sign_big triang	Msbt
Decoration	Motifs pointed signs along main lines of decor	Mps
Decoration	Motifs motive-horizontal rows	Mhr
Decoration	Motifs horiz rows-groups-empty field	Mhrg
Decoration	Motifs vertical	Mvr
Decoration	Motifs oblique parallel	Mor
Decoration	Motifs oblique-put at angle rows	Mop
Decoration	Motifs groups oblique (Middle Don style)	Mgo
Decoration	Motifs impr-zigzag horiz	Mzh
Decoration	Motifs impr-zigzag vertical	Mzv
Decoration	Motifs geometrical des	Mg
Decoration	Motifs wavy	Mm
Decoration	Motifs triangle filled	Mtf
Decoration	Motifs zigzag line	Mzl
Decoration	Motifs net	Mn
Decoration	Motifs triangle pendant	Mtp
Decoration	Motifs oval figures filled	Mof
Decoration	Motifs dense meshed net	Mdm
Decoration	Motifs pits+oblique stamp	Mpo
Decoration	Motifs rocking chair	Mtrc
Decoration	Motifs retreating	Mtr
Decoration	Motifs empty fields	Mg

Table	Description	Code
Decoration	Motifs figure/grouped	Mf
Decoration	Location row under rim	Lr
Decoration	Location rim-outer	Lro
Decoration	Location rim edge	Lre
Decoration	Location rim inside	Lri
Decoration	Location all	La
Decoration	holes under rim	H
Decoration	quadrangle from inside (Lys gora)-pearl outside under rim	Q
Decoration	depressions-pearls inside under rim	DP
Decoration	depressions-no pearls	DnP
Decoration	depr-pearls outside	DPo
Decoration	groove under the rim	Gr

Supplementary Table 7: 95% confidence interval for Mantel correlation coefficients and associated *p*-values

	Circuitscape Resistance	Modelled space-time distance	Great circle distance	Organic Residues	Technology	Decoration	Morphology
Circuitscape Resistance		**0.42 – 0.54**	**0.17 – 0.29**	-0.09 – -0.01	-0.06 – 0.03	-0.02 – 0.04	-0.02 – 0.03
Modelled space-time distance	**0.001**		**0.28 – 0.37**	-0.11 – -0.06	0.08 – 0.14	0.02 – 0.08	0.02 – 0.08
Great circle distance	**0.002**	**0.001**		-0.01 – 0.04	**0.22 – 0.28**	**0.09 – 0.14**	**0.15 – 0.2**
Organic Residues	0.377	0.1	0.77		**0.15 – 0.22**	**0.11 – 0.17**	**0.1 – 0.16**
Technology	0.777	0.057	**0.001**	**0.001**		**0.27 – 0.33**	**0.3 – 0.36**

Decoration	0.912	0.289	**0.002**	**0.001**	**0.001**		**0.3 – 0.36**
Morphology	0.193	0.267	**0.001**	**0.001**	**0.001**	**0.001**	

NB: Upper diagonal contains the 95% confidence interval for Mantel correlation coefficient (*r*) lower diagonal contains associated *p*-values (two-tailed, null hypothesis: r = 0, lower diagonal); highly significant correlations marked in bold type.

Supplementary Table 8: Distribution of Jaccard distances (interquartile range)

	Organic residues	Technology	Morphology	Decoration
All sites	0.4 – 0.8	0.7 – 0.8	0.8 – 1	0.8 – 1
Baltic	0.3 – 0.8	0.4 – 0.7	0.7 – 1	0.7 – 1
E. Forests and steppe	0.2 – 0.6	0.4 – 0.6	0.5 – 0.9	0.6 – 0.8
Taiga	0.3 – 0.8	0.5 – 0.9	0.8 – 1	0.7 – 1
W. forests and steppe	0.3 – 0.6	0.5 – 0.7	0.8 – 1	0.6 – 0.9

NB: The scale is 0 (identical) to 1 (completely different).

Supplementary Table 9: Mantel test coefficients with vessels as the unit of analysis

	Organic residues	Technology	Decoration
Technology	0.28		
Decoration	0.12	0.25	
Form	0.14	0.36	0.10

Supplementary Table 10: Mantel test coefficients limited to major regions

	Don-Volga region (41 sites)				Ukraine (28 sites)			
	GC Distance	Organic residues	Tech.	Decor.	GC Distanc e	Organic residues	Tech.	Decor.
Organic residues	–0.14				0.05[a]			
Tech.	0.15[a] p=0.09	0.44			0.26	0.22		
Decor.	–0.001[a]	0.43	0.45		0.13[a] p=0.14	–0.005[a]	0.20	
Morph.	0.19	0.17	0.34	0.21	0.07[a]	0.18	0.26	0.25

[a] – not significant (p value indicated for higher coefficients)

Supplementary Table 11 : Mantel test coefficients stratified by vegetation zone

	Steppe (50 sites)		Forest (37 sites)		Taiga (10 sites)	
	GC Distance	Organic residues	GC Distance	Organic residues	GC Distance	Organic residues
Organic residues	0.01[a]		0.01[a]		0.02[a]	
Technology	0.18	0.32	0.30	0.37	-0.02[a]	0.25[a] p=0.08
Decoration	0.15	0.24	0.11	0.21	0.45	0.10[a] p=0.25
Morphology	0.18	0.21	0.23	0.26	-0.06[a]	0.41

[a] – not significant (p value indicated for higher coefficients)

Supplementary Table 12 : Mantel test coefficients for earliest forms only

	GC Distance	Residues holding GC distance	Residues	Technology	Decoration
Organic residues	-0.02[a]				
Technology	0.23	0.27	0.26		
Decoration	0.09	0.16	0.16	0.27	
Morphology	0.18	0.16	0.16	0.31	0.31

[a] – not significant

Supplementary References

1. Silva, F. & Steele, J. New methods for reconstructing geographical effects on dispersal rates and routes from large-scale radiocarbon databases. *J. Archaeol. Sci.* **52**, 609–620 (2014).
2. Silva, F. & Steele, J. Modeling Boundaries Between Converging Fronts In Prehistory. *Adv. Complex Syst.* **15**, 1150005 (2012).
3. Jordan, P. *et al.* Modelling the diffusion of pottery technologies across Afro-Eurasia: emerging insights and future research. *Antiquity* **90**, 590–603 (2016).
4. Bronk Ramsey, C. Bayesian analysis of radiocarbon dates. *Radiocarbon* **51**, 337–360 (2009).
5. Grechkina, T. Y. Vybornov, A. A. Lebedev, Y. S. ЖИЛИЩЕ РАННЕНЕОЛИТИЧЕСКОЙ СТОЯНКИ БАЙБЕК В СЕВЕРНОМ ПРИКАСПИИ (The dwelling at the early Neolithic site of Baybek in the Northern Caspian region). *КРАТКИЕ СООБЩЕНИЯ ИНСТИТУТА АРХЕОЛОГИИ Институт археологии РАН (Brief Reports of the Institute of Archaeology, RAS Moscow)* **262**, 142–155 (2021).
6. Vybornov, A. A. *et al.* Results of excavations at the Algay site in the steppe Volga region in 2020. *ISSCHC* **3**, 100–121 (2021).
7. Bronk Ramsey, C. Dealing with outliers and offsets in radiocarbon dating. *Radiocarbon* **51**, 1023–1045 (2009).
8. Kulkova, M. A., Vybornov, A. A., Yudin, A., Doga, N. & Popov, A. New interdisciplinary research on Neolithic-Eneolithic sites in the Low Volga River region. *Doc. Praehist.* **46**, 376–387 (2019).
9. Yudin, A. I. *et al.* Neolithic site Algay in Low Povolzhye. *Samara Journal of Science* **5**, 61–68 (2016).
10. Courel, B. *et al.* The use of early pottery by hunter-gatherers of the Eastern European forest-steppe. *Quat. Sci. Rev.* **269**, 107143 (2021).
11. Bondetti, M. *et al.* Neolithic farmers or Neolithic foragers? Organic residue analysis of early pottery from Rakushechny Yar on the Lower Don (Russia). *Archaeol. Anthropol. Sci.* **13**, 141 (2021).

12. Bondetti, M. *et al.* Fruits, fish and the introduction of pottery in the Eastern European plain: Lipid residue analysis of ceramic vessels from Zamostje 2. *Quat. Int.* (2019) doi:10.1016/j.quaint.2019.05.008.

13. Meadows, J. *et al.* Absolute chronology of Upper Volga-type pottery: More evidence from Zamostje 2. *Samara Journal of Science* **4**, 113–121 (2015).

14. Courel, B. *et al.* Organic residue analysis shows sub-regional patterns in the use of pottery by Northern European hunter-gatherers. *R Soc Open Sci* **7**, 192016 (2020).

15. Liiva, A. & Loze, I. Mesolithic and Neolithic Habitation of the Eastern Baltic. *Radiocarbon* **35**, 503–506 (1993).

16. Liiva, A. & Loze, I. Tartu Radiocarbon Dates XIII. *Radiocarbon* **36**, 153–158 (1994).

17. Loze, I. The Early Neolithic in Latvia. The Narva Culture. *Acta Archaeol.* **63**, 119–140 (1992).

18. Vybornov, A. *et al.* Traits of the Neolithic-Eneolithic archaeological layers' formation at the sites of Algay and Oroshaemoe in the low Volga basin (Low Povoljie). *Acta Geographica Lodziensia* **110**, 49–59 (2020).

19. Librado, P. *et al.* The Evolutionary Origin and Genetic Makeup of Domestic Horses. *Genetics* **204**, 423–434 (2016).

20. Vybornov, A. A. *et al.* About ancient ceramic traditions of the population of the northern Caspian region. *VOLGOGRADSKII GOSUDARSTVENNYI UNIVERSITET-VESTNIK-SERIYA 4-ISTORIYA REGIONOVEDENIE MEZHDUNARODNYE OTNOSHENIYA* **25**, 141–151 (2020).

21. Dolbunova, E. *et al.* Rakushechny Yar site: lacustrine and fluvial deposits, buried soils and shell platforms from 6th mill. BC. *Acta Geographica Lodziensia* **110**, 61–80 (2020).

22. Lozovski, V. M., Lozovskaya, O. V., Zaitceva, G. I. & Possnert, G. Комплекс верхневолжской керамики ранненеолитического слоя стоянки Замостье 2: типологический состав и хронологические рамки (Early …. *Самарский научный* (2014).

23. ASTER Global Digital Elevation Map. https://asterweb.jpl.nasa.gov/gdem.asp.

24. GRASS Development Team. *Geographic Resources Analysis Support System (GRASS) Software,*

Version 7.0. Open Source Geospatial Foundation. (2015).

25. Legendre, P. & Oksanen, M. J. Package 'lmodel2'. *See https://CRAN. R-project. org/package= lmodel2* (2018).
26. Pinhasi, R., Fort, J. & Ammerman, A. J. Tracing the Origin and Spread of Agriculture in Europe. *PLoS Biol.* **3**, e410 (2005).
27. Bocquet-Appel, J.-P., Naji, S., Vander Linden, M. & Kozlowski, J. Understanding the rates of expansion of the farming system in Europe. *J. Archaeol. Sci.* **39**, 531–546 (2012).
28. Isern, N., Zilhão, J., Fort, J. & Ammerman, A. J. Modeling the role of voyaging in the coastal spread of the Early Neolithic in the West Mediterranean. *Proc. Natl. Acad. Sci. U. S. A.* **114**, 897–902 (2017).
29. Fort, J. & Pareta, M. M. Long-distance dispersal effects and Neolithic waves of advance. *J. Archaeol. Sci.* (2020).
30. Fort, J., Mercè Pareta, M. & Sørensen, L. Estimating the relative importance of demic and cultural diffusion in the spread of the Neolithic in Scandinavia. *J. R. Soc. Interface* **15**, 20180597 (2018).
31. Russell, T., Silva, F. & Steele, J. Modelling the Spread of Farming in the Bantu-Speaking Regions of Africa: An Archaeology-Based Phylogeography. *PLoS One* **9**, e87854 (2014).
32. Jerardino, A., Fort, J., Isern, N. & Rondelli, B. Cultural Diffusion Was the Main Driving Mechanism of the Neolithic Transition in Southern Africa. *PLoS One* **9**, e113672 (2014).
33. Bryant, D. & Moulton, V. Neighbor-net: an agglomerative method for the construction of phylogenetic networks. *Mol. Biol. Evol.* **21**, 255–265 (2004).
34. Wood, S. N. Thin plate regression splines. *J. R. Stat. Soc. Series B Stat. Methodol.* **65**, 95–114 (2003).

`
