# Supplementary material for: The transmission of pottery technology among prehistoric European hunter-gatherers
Source: Nat Hum Behav. 2022 Dec 22;7(2):171–83. doi: 10.1038/s41562-022-01491-8 (PMC9957732; doi:10.1038/s41562-022-01491-8)
Supplement: Supplementary file 2 — Reporting Summary [file 41562_2022_1491_MOESM2_ESM.pdf]

nature portfolio

Corresponding author(s): T. Rowan McLaughlin
Last updated by author(s): Sep 5, 2022

Reporting Summary

Nature Portfolio wishes to improve the reproducibility of the work that we publish. This form provides structure for consistency and transparency in reporting. For further information on Nature Portfolio policies, see our Editorial Policies and the Editorial Policy Checklist.

Statistics

For all statistical analyses, confirm that the following items are present in the figure legend, table legend, main text, or Methods section.

n/a	Confirmed	
☐	☒	The exact sample size (*n*) for each experimental group/condition, given as a discrete number and unit of measurement
☐	☒	A statement on whether measurements were taken from distinct samples or whether the same sample was measured repeatedly
☐	☒	The statistical test(s) used AND whether they are one- or two-sided<br>*Only common tests should be described solely by name; describe more complex techniques in the Methods section.*
☐	☒	A description of all covariates tested
☐	☒	A description of any assumptions or corrections, such as tests of normality and adjustment for multiple comparisons
☐	☒	A full description of the statistical parameters including central tendency (e.g. means) or other basic estimates (e.g. regression coefficient) AND variation (e.g. standard deviation) or associated estimates of uncertainty (e.g. confidence intervals)
☐	☒	For null hypothesis testing, the test statistic (e.g. *F*, *t*, *r*) with confidence intervals, effect sizes, degrees of freedom and *P* value noted<br>*Give P values as exact values whenever suitable.*
☐	☒	For Bayesian analysis, information on the choice of priors and Markov chain Monte Carlo settings
☐	☒	For hierarchical and complex designs, identification of the appropriate level for tests and full reporting of outcomes
☐	☒	Estimates of effect sizes (e.g. Cohen's *d*, Pearson's *r*), indicating how they were calculated

Our web collection on statistics for biologists contains articles on many of the points above.

Software and code

Policy information about availability of computer code

Data collection	MSD Chemstation version F.01.03.2357; Mass Hunter version B.07.01; NIST MS search version 2.2; Isodat version 3.0; IonOS version 4; Google Sheets
Data analysis	R version 4.0 (with packages vegan 2.5.7, ecodist 2.0.7, lmodel2 1.7.2, dplyr 1.0, scatterpie 0.18, and their dependencies); custom scripts (available at doi.org/10.5281/zenodo.6619101); GRASS GIS version 7.4; SAGA GIS version 7; Julia version 1.6; circuitscape version 5; IsoMemo version 1.9; OxCal version 4.4

For manuscripts utilizing custom algorithms or software that are central to the research but not yet described in published literature, software must be made available to editors and reviewers. We strongly encourage code deposition in a community repository (e.g. GitHub). See the Nature Portfolio guidelines for submitting code & software for further information.

Data

Policy information about availability of data

All manuscripts must include a data availability statement. This statement should provide the following information, where applicable:

- Accession codes, unique identifiers, or web links for publicly available datasets
- A description of any restrictions on data availability
- For clinical datasets or third party data, please ensure that the statement adheres to our policy

Data files including all the ceramic data and contingency tables for the organic residue traits are contained in an electronic repository accessed via the following URL: doi.org/10.5281/zenodo.6619101

Human research participants

Policy information about studies involving human research participants and Sex and Gender in Research.

Reporting on sex and gender	N/A
Population characteristics	N/A
Recruitment	N/A
Ethics oversight	NA

Note that full information on the approval of the study protocol must also be provided in the manuscript.

Field-specific reporting

Please select the one below that is the best fit for your research. If you are not sure, read the appropriate sections before making your selection.

☒ Life sciences ☐ Behavioural & social sciences ☐ Ecological, evolutionary & environmental sciences

For a reference copy of the document with all sections, see nature.com/documents/nr-reporting-summary-flat.pdf

Life sciences study design

All studies must disclose on these points even when the disclosure is negative.

Sample size	Sample size was determined by the quantity of archaeological remains curated in museum collections for the cultural groups under analysis.
Data exclusions	No data were excluded from initial analyses; samples that failed to yield lipid concentrations below a predetermined analytical minimum of 5 µg per g of potsherds and 100 µg per g for charred surface deposits were excluded from trait-based analyses.
Replication	Samples for mass spectrometry were not measured in duplicate but replicated measurements of a lab standard were run routinely with each batch of samples.
Randomization	Samples were classified by geographical region considering the location of the archaeological sites.
Blinding	Blinding was not relevant for our study; some of the samples were selected by their morphological and decorative features.

Reporting for specific materials, systems and methods

We require information from authors about some types of materials, experimental systems and methods used in many studies. Here, indicate whether each material, system or method listed is relevant to your study. If you are not sure if a list item applies to your research, read the appropriate section before selecting a response.

Materials & experimental systems

n/a	Involved in the study
☒	☐ Antibodies
☒	☐ Eukaryotic cell lines
☐	☒ Palaeontology and archaeology
☒	☐ Animals and other organisms
☒	☐ Clinical data
☒	☐ Dual use research of concern

Methods

n/a	Involved in the study
☒	☐ ChIP-seq
☒	☐ Flow cytometry
☒	☐ MRI-based neuroimaging

Palaeontology and Archaeology

Specimen provenance	Information on specimen provenance, including site location, is detailed in the paper, including contextual details for new radiocarbon dates.
Specimen deposition	Pottery samples were destroyed during the organic residue analyses
Dating methods	New radiocarbon dates were obtained using terrestrial samples (herbivore bones, wood charcoal, plant macrofossils) found in association with the samples in this study, and used to develop chronological models for a select number of sites using OxCal 4.4 and the IntCal20 calibration dataset.

☒ Tick this box to confirm that the raw and calibrated dates are available in the paper or in Supplementary Information.

Ethics oversight	No new archaeological excavations were performed so ethical approval was not required. Permission for destructive analysis was given by the institutions curating the material.

Note that full information on the approval of the study protocol must also be provided in the manuscript.
